# BCLAF1 links RNA splicing to ATF4-dependent metabolic adaptation in acute myeloid leukemia

**DOI:** 10.1126/sciadv.aeg0571

**Published:** 2026-11-04

**Authors:** Laura López-Hernández, Stephanie J. Crowley, Sara Cea-Sánchez, Ricardo de Arellano, Neetij Krishnan, Patrick Toolan-Kerr, Lynn White, Domenico Ignoti, Emily Soto-Hidalgo, Stefano Gustincich, Román González-Prieto, Luca Pandolfini, Isaia Barbieri, Patricia Altea-Manzano, Jeffrey A. Magee, Francesca Ferraro, Samuel J. Taylor, Jeffrey J. Bednarski, Gonzalo Millán-Zambrano

**Affiliations:** ^1^ Centro Andaluz de Biología Molecular y Medicina Regenerativa-CABIMER, CSIC-Universidad de Sevilla-Universidad Pablo de Olavide, 41092 Seville, Spain.; ^2^ Departamento de Genética, Universidad de Sevilla, 41012 Seville, Spain.; ^3^ Department of Pediatrics, Washington University School of Medicine, St. Louis, MO.; ^4^ Department of Molecular Biotechnology and Health sciences, Molecular Biotechnology Center, University of Turin, Turin, Italy.; ^5^ Center for Human Technologies, Istituto Italiano di Tecnologia (IIT), Genova, Italy.; ^6^ Departamento de Biología Celular, Universidad de Sevilla, 41012 Seville, Spain.; ^7^ Department of Internal Medicine, Division of Oncology, Washington University School of Medicine, St. Louis, MO.; ^8^ Department of Pediatrics, University of Pittsburgh School of Medicine, Pittsburgh, PA.

## Abstract

Acute myeloid leukemia (AML) is driven by genetic alterations and non-mutational mechanisms that disrupt normal hematopoiesis and support leukemic cell survival. While the mutational landscape of AML is well characterized, the non-genetic processes sustaining leukemic maintenance remain less understood. Using human AML cells and murine models, we identify BCL2-associated transcription factor 1 (BCLAF1) as a key regulator of leukemic progression through control of mRNA processing. BCLAF1 physically associates with core spliceosome components and regulates alternative splicing programs in leukemic cells. Mechanistically, BCLAF1 is required for productive splicing of activating transcription factor 4 (ATF4) mRNA, thereby sustaining ATF4 protein levels and downstream metabolic gene expression programs. Loss of BCLAF1 reduces ATF4 levels, leading to impaired de novo amino acid biosynthesis and increased sensitivity to venetoclax. Together, these findings uncover a previously unrecognized mechanism linking RNA splicing control to metabolic adaptation in AML, identifying BCLAF1 as a potential therapeutic vulnerability.

## INTRODUCTION

Acute myeloid leukemia (AML) is an aggressive hematologic malignancy characterized by the clonal expansion of immature myeloid precursor cells. While many patients initially respond to chemotherapy, most eventually relapse due to the persistence of drug-resistant leukemic stem cells (LSCs) (*1*, *2*). Despite recent therapeutic advances, AML remains largely incurable, with a 5-year overall survival rate of about 20% (*3*), underscoring the need for deeper mechanistic insights and new therapeutic targets (*4*).

AML is a biologically heterogeneous disease driven by a wide array of somatic oncogenic mutations. Among these, mutations in spliceosome genes, present in 10–25% of cases, are particularly prominent (*5*, *6*). Although their precise oncogenic roles remain incompletely understood, growing evidence implicates aberrant mRNA splicing as a critical mediator of leukemogenesis (*7*, *8*). Notably, AML cells bearing spliceosome mutations exhibit selective sensitivity to perturbations of the splicing machinery (*9*–*12*), highlighting the spliceosome as a promising therapeutic target.

By contrast, the non-mutational molecular processes that sustain AML are less well defined. One such process is metabolic reprogramming, which has recently emerged as a hallmark of leukemogenesis (*13*, *14*). LSCs exhibit elevated amino acid uptake and catabolism to support oxidative phosphorylation (*15*). In addition, AML cells display specific dependencies on distinct amino acids (*16*–*21*), underscoring the therapeutic potential of targeting amino acid metabolism. However, the upstream regulatory mechanisms that coordinate these metabolic adaptations remain poorly defined.

Emerging evidence indicates that mRNA splicing and cellular metabolism are interconnected. Metabolic cues extensively regulate mRNA splicing programs, enabling rapid cellular adaptation to nutrient availability and stress (*22*). Conversely, recent work has shown that perturbation of the splicing machinery can reshape cellular metabolic states, including in myeloid malignancies (*23*–*27*). However, the molecular mechanisms linking splicing regulation to metabolic reprogramming in AML remain incompletely defined.

Here, we uncover a central role for BCLAF1 in coupling RNA processing with amino acid metabolic programs in AML. We show that BCLAF1 promotes productive mRNA splicing in AML cells and identify the metabolic regulator ATF4 as a key downstream effector. Loss of BCLAF1 disrupts *ATF4* mRNA processing, leading to reduced ATF4 protein levels and down-regulation of ATF4-dependent amino acid metabolic genes. These findings establish BCLAF1 as a crucial regulator of mRNA processing in AML and reveal a mechanistic link between splicing control and amino acid metabolic adaptation supporting leukemic cell survival.

## RESULTS

### BCLAF1 supports AML pathogenesis

Based on our previous work identifying BCLAF1 as a context-dependent regulator of hematopoietic progenitor–associated transcriptional programs (*28*), we explored whether BCLAF1 might also contribute to leukemic cell states in AML. To assess the clinical relevance of BCLAF1 in human cancer, we analyzed publicly available gene expression data from The Cancer Genome Atlas (TCGA). BCLAF1 is most highly expressed in AML samples compared to other cancer types (Fig. 1A) and is significantly elevated in AML relative to healthy hematopoietic tissues (Fig. 1B), suggesting that BCLAF1 may play a central role in AML. To directly assess this possibility, we performed growth competition assays in two independent human *MLL-AF9* rearranged AML cell lines, MOLM-13 (*FLT3-ITD*) and THP-1 (*FLT3* wild-type). Cells were transduced with constructs co-expressing GFP, Cas9, and either a non-targeting negative control sgRNA, one of two independent *BCLAF1*-targeting sgRNAs, or a positive control sgRNA against *RBM39*, which is essential for AML maintenance (*29*). In both cell lines, we observed a substantial depletion of GFP-positive cells expressing *BCLAF1* sgRNAs (Fig. 1C), indicating that BCLAF1 is required for human AML cell proliferation.

**Fig. 1.**
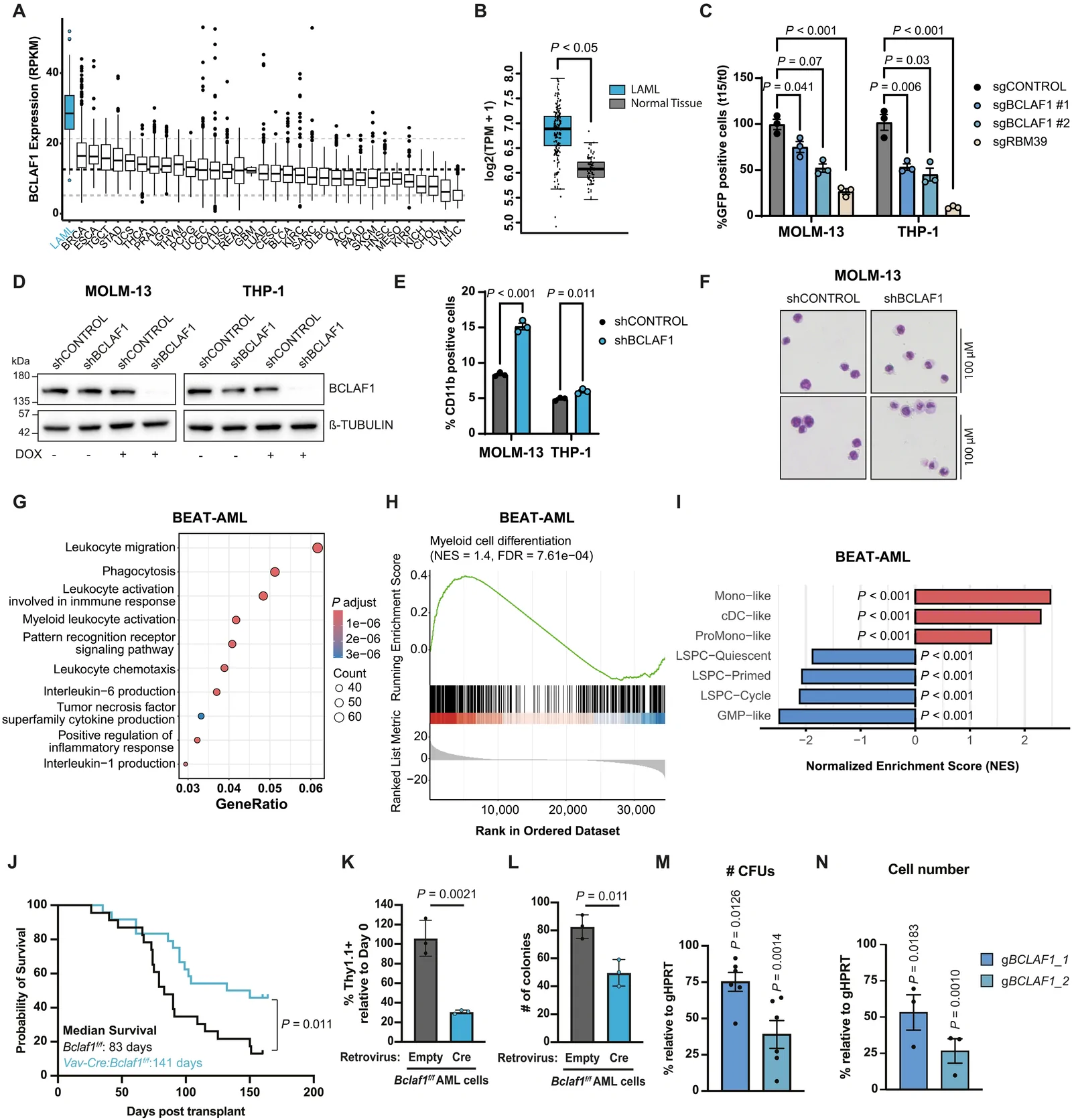
BCLAF1 supports AML pathogenesis. (**A**) BCLAF1 expression across TCGA cancer types. (**B**) BCLAF1 expression in LAML patient samples (n = 173) and normal hematopoietic tissues (n = 70). (**C**) Growth competition assays showing the percentage of GFP-positive cells from days 2–15 after infection with LentiCRISPRv2GFP constructs expressing the indicated gRNAs. (**D**) Immunoblot analysis of BCLAF1 in shCONTROL and shBCLAF1 cells treated with or without DOX for 96 hours. β-tubulin was used as loading control. (**E**) Flow cytometry analysis showing percentage of CD11b-positive cells following treatment as in D. (**F**) Representative Wright–Giemsa staining of cells treated as in D. Scale bar, 100 μm; original magnification, 10×. (**G**) GO terms enriched among genes up-regulated in BCLAF1-low versus BCLAF1-high BEAT-AML samples. (**H** and **I**) GSEA of myeloid cell differentiation (H) and AML hierarchy signatures (I) in BCLAF1-low versus BCLAF1-high BEAT-AML samples. (**J**) Kaplan-Meier survival curve of mice transplanted with MLL-AF9 expressing *Bclaf1^f/f^* (n = 23) or *Vav-Cre:Bclaf1^f/f^* (n = 24) HSPCs. Schematic in fig. S2A. Inset shows median survival. (**K**) Primary *Bclaf1^f/f^* AMLs isolated from mice in J were transduced with empty or Cre-expressing vectors and expanded in mixed co-culture with non-transduced cells. Bars show transduced Thy1.1+ cells at day 7 relative to day 0. (**L**) Thy1.1+ cells from K were sorted and plated in MethoCult; CFUs were quantified at day 7. (**M** and **N**) Percentage of CFUs (M) and relative cell recovery (N) of primary human AML samples targeted with Cas9 and two independent *BCLAF1* gRNAs normalized to *HPRT*-targeted control cells. Data are mean ± SEM [(C) and (E)] or mean ± SD [(K) to (N)] of 3 biological replicates and with statistics by either unpaired [(C), (E), (K), and (L)] or paired *t* test [(M) and (N)].

To investigate the direct effects of BCLAF1 depletion, we generated MOLM-13 and THP-1 stable cell lines expressing a doxycycline (DOX)-inducible shRNA targeting *BCLAF1* (shBCLAF1) or a non-targeting control (shCONTROL). Upon DOX treatment, both cell lines showed efficient BCLAF1 depletion (Fig. 1D) and reduced proliferation (fig. S1A), confirming a functional requirement for BCLAF1 in AML cells. To further characterize the cellular consequences of BCLAF1 loss, we assessed the surface expression of CD11b, a marker associated with myeloid differentiation, and apoptosis levels using annexin V staining. We found that depletion of BCLAF1 increases CD11b expression in both MOLM-13 and THP-1 cells (Fig. 1E). In line with this, Wright–Giemsa staining revealed morphological alterations, including increased asymmetry and cytoplasmic volume (Fig. 1F). Comparatively, only modest changes in apoptosis were observed (fig. S1B). Similar results were obtained using a second independent shRNA targeting *BCLAF1* (fig. S1, C, D, and E), suggesting that BCLAF1 loss is associated with a shift toward a less immature leukemic state.

To assess whether BCLAF1 expression correlates with leukemic states in AML patients, we stratified BEAT-AML (*30*) cohort samples based on *BCLAF1* mRNA expression levels and defined the top 25% (145/578) as *BCLAF*1-high cases, and the bottom 25% (145/578) as *BCLAF1*-low cases. In total, we identified 3968 differentially expressed genes (false discovery rate (FDR) ≤ 0.05, foldchange (FC) ≥ 2) between these sets of samples (table S1), including 1527 up-regulated transcripts in *BCLAF1*-low cases. Gene ontology analysis of up-regulated genes in *BCLAF1*-low patients showed an enrichment of categories such as leukocyte migration (GO:0050900) and myeloid leukocyte activation (GO:0002274) (Fig. 1G), suggestive of a less immature leukemic state. In line with this, gene set enrichment analysis identified a marked upregulation of myeloid cell differentiation-related genes in BCLAF1-low patients (Fig. 1H). To determine whether BCLAF1 expression was associated with specific leukemic cell-state programs, we used established AML hierarchy gene expression signatures (*31*, *32*). Notably, gene set enrichment analysis showed that BCLAF1-low patients were depleted for primitive/progenitor-associated programs, including leukemic stem and progenitor cell (LSPC)-quiescent, LSPC-primed, LSPC-cycling and granulocyte-macrophage progenitor (GMP)-like signatures, and enriched for more mature myeloid-like programs, including Mono-like, conventional dendritic cell (cDC)-like and ProMono-like signatures (Fig. 1I). Similar, although weaker, trends were observed in the pediatric TARGET-AML cohort (fig. S1, F, G, and H). Taken together, these data support an association between BCLAF1 expression and primitive leukemic transcriptional programs in primary AML.

Next, we sought to determine whether loss BCLAF1 impairs of AML pathogenesis in vivo. Retroviral expression of the MLL-AF9 fusion gene in murine hematopoietic stem and progenitor cells (HSPCs) results in an established, aggressive form of AML (*33*, *34*). We used this model of primary AML to assess the role of BCLAF1 in leukemic initiation in vivo. HSPCs from adult (8-week-old) donor CD45.2^+^ wild-type *Bclaf1^f/f^* or *Vav-Cre:Bclaf1^f/f^* mice, which have selective deletion of *Bclaf1* in hematopoietic cells (*28*), were transduced with retrovirus co-expressing an MLL-AF9 fusion construct and a GFP reporter, then transplanted into lethally-irradiated CD45.1^+^ recipient mice (fig. S2A). Leukemic burden was monitored by percentage of GFP^+^ cells in peripheral blood. Of the mice that received MLL-AF9-transduced wild-type *Bclaf1^f/f^* HSPCs, 87% (20 of 23) developed AML with a median survival of 83 days (Fig. 1J and fig. S2B). In contrast, only 54% (13 of 24) of mice receiving MLL-AF9-transduced *Vav-Cre:Bclaf1^f/f^* HSPCs developed leukemia with a median survival of 141 days (Fig. 1J). The surviving mice that received MLL-AF9-transduced *Vav-Cre:Bclaf1^f/f^* HSPCs had no evidence of AML (defined by absence of GFP+ cells) in bone marrow and spleen at 5 months post-transplant (fig. S2C). These results indicate that loss of BCLAF1 impairs AML initiation in vivo, as evidenced by a reduced incidence of leukemic transformation and prolonged survival in mice receiving *Bclaf1*-deficient MLL-AF9-expressing HSPCs.

To further investigate whether loss of BCLAF1 compromises AML expansion capacity, we cultured wild-type MLL-AF9-expressing *Bclaf1^f/f^* leukemias in vitro and transduced them with retrovirus expressing Cre recombinase to inactivate the *Bclaf1* allele. Deletion of *Bclaf1* led to impaired expansion of AML cells, as indicated by a more substantial loss of cells expressing Cre recombinase (Thy1.1^+^ cells) compared with control cells expressing empty viral vector (Fig. 1K). Additionally, sorted Cre-expressing *Bclaf1^f/f^* AML cells generated fewer colonies compared to sorted control *Bclaf1*-sufficient AML cells (Fig. 1L), indicating that BCLAF1 is required for AML maintenance ex vivo.

To extend our findings to primary human AML, we performed CRISPR/Cas9-mediated *BCLAF1* targeting in primary AML samples collected from bone marrow or peripheral blood of three independent patients with distinct genetic backgrounds (details in figure legend). Cells were transfected with Cas9/gRNA ribonucleoprotein (RNP) complexes targeting either hypoxanthine phosphoribosyltransferase (*HPRT*) as a control or *BCLAF1* using two independent gRNAs and subsequently plated in MethoCult for colony-forming assays. *BCLAF1* targeting markedly impaired colony formation and reduced overall cell number compared with control *HPRT* (Fig. 1, M and N). These effects were consistently observed across all three genetically distinct primary AML samples (fig. S2, D, E, F, G, H, and I), supporting a functional requirement for BCLAF1 in primary AML cell growth and clonogenic potential. Together, these findings extend our observations to primary human AML and further support an important role for BCLAF1 in AML pathogenesis.

### BCLAF1 regulates mRNA splicing in AML cells

BCLAF1 is a multifunctional protein implicated in diverse biological processes (*35*–*40*). To gain mechanistic insight into BCLAF1 function in AML, we sought to identify its interacting partners by performing immunoprecipitation followed by mass spectrometry (IP-MS) in MOLM-13 cells. To enable specific pulldown of the protein, we transduced cells with a lentiviral construct expressing an ALFA-tagged (*41*) version of BCLAF1. Immunoblot analysis confirmed that ALFA-BCLAF1 was expressed at levels comparable to endogenous protein, ruling out potential overexpression artifacts (fig. S3A). IP-MS analysis identified 19 BCLAF1-specific interactors (Fig. 2A and table S3), which form a highly connected protein network enriched in components of the spliceosome complex (fig. S3B). Consistently, gene ontology analysis of BCLAF1-interactors indicated a strong enrichment for mRNA splicing and processing categories (Fig. 2B), supporting a functional interaction between BCLAF1 and the splicing machinery in AML cells.

**Fig. 2.**
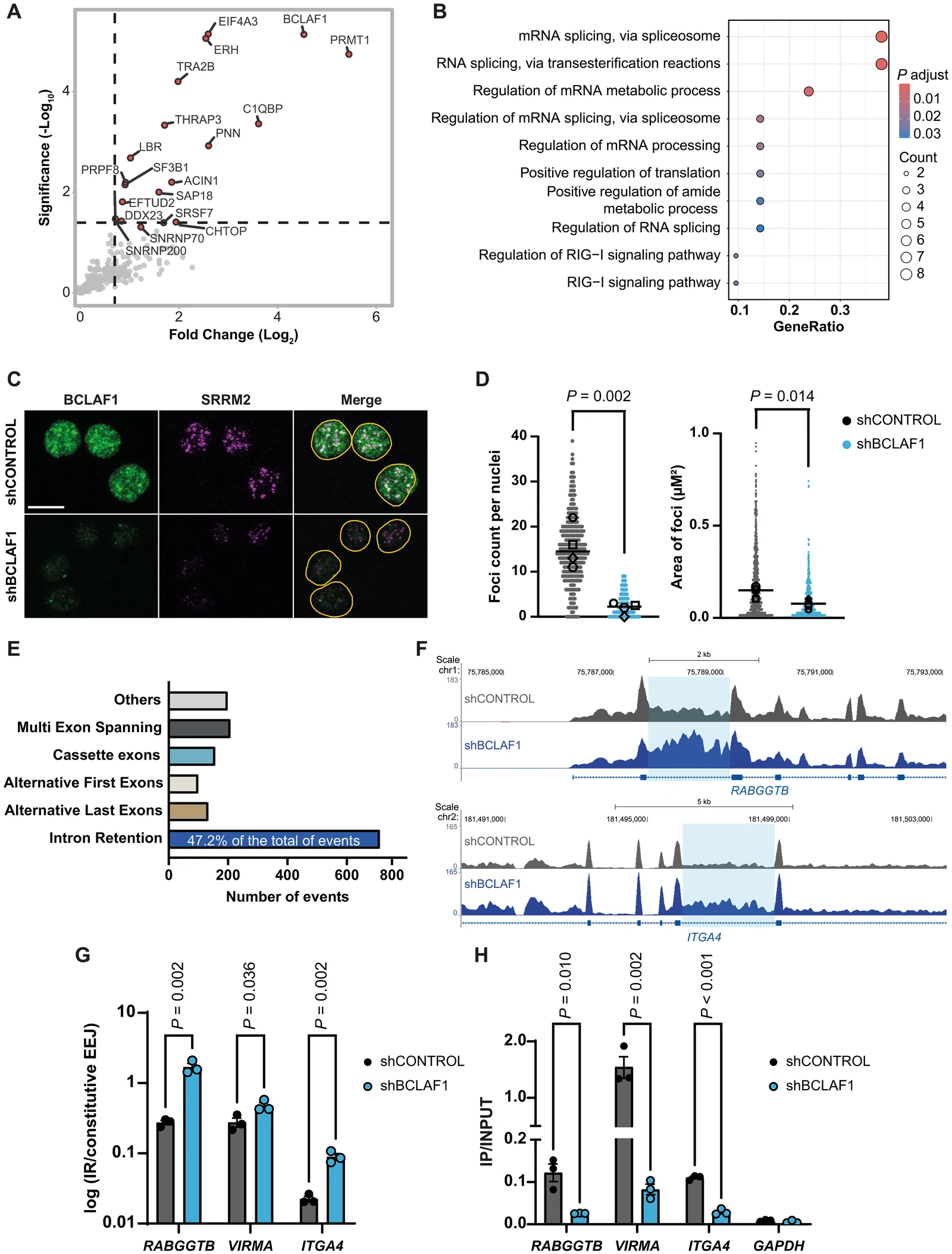
BCLAF1 regulates mRNA splicing in AML cells. (**A**) Volcano plot showing BCLAF1-interacting proteins identified by immunoprecipitation followed by mass spectrometry (IP-MS). The *x*-axis indicates log2 fold change (ALFA-BCLAF1 IP vs. control), and the *y*-axis shows –log10 *P* value. Significant interactors (fold change ≥1.5, *P* value ≤0.05) are highlighted in red. (**B**) Dot plot of GO terms enriched among BCLAF1 interactors. (**C**) Immunofluorescence analysis of BCLAF1 and the nuclear speckle marker SRRM2 in shCONTROL and shBCLAF1 MOLM-13 cells treated with DOX for 96 hours. Yellow lines mark the nuclear boundary (FIJI mask). Scale bar, 10 μm. (**D**) Quantification of SSRM2 foci size and number per nucleus in shCONTROL and shBCLAF1 MOLM-13 cells treated with DOX for 96 hours. Data represent the median values from four independent biological replicates, each shown as a distinct symbol. Statistical significance was assessed using the mean of medians and a Mann-Whitney test. (**E**) Bar graph showing the distribution of alternative splicing event types significantly altered upon BCLAF1 depletion (96 hours of DOX treatment) as quantified by MAJIQ. (**F**) Representative genome browser snapshots of RNA-seq data from shCONTROL and shBCLAF1 MOLM-13 cells treated with DOX for 96 hours. (**G**) RT-qPCR experiments showing relative intron retention (IR) levels of the indicated transcripts in shCONTROL and shBCLAF1 MOLM-13 cells treated with DOX for 96 hours. Data represent mean ± SEM of three biological replicates. Statistics by unpaired *t* test. (**H**) RIP-qPCR experiments showing BCLAF1 association with the indicated transcripts in shCONTROL and shBCLAF1 MOLM-13 cells treated with DOX for 96 hours. *GAPDH* was used as a negative control. Data represent mean ± SEM of three biological replicates. Statistics by unpaired *t* test.

Given that BCLAF1 cellular distribution varies across cancer types (*35*–*38*), we next examined its localization in MOLM-13 cells by immunofluorescence analysis. We observed that endogenous BCLAF1 localizes to dot-like structures throughout the nucleus that are reminiscent of nuclear speckles (Fig. 2C), subnuclear compartments enriched in splicing factors (*42*). Co-immunostaining with the nuclear speckle marker serine/arginine repetitive matrix 2 (SRRM2) (*43*) confirmed partial colocalization (Fig. 2C), indicating that BCLAF1 is associated with nuclear speckles in AML cells. Interestingly, depletion of BCLAF1 reduced both the size and number of nuclear speckles (Fig. 2, C and D), suggesting that BCLAF1 contributes to nuclear speckle integrity. In support of this, gene set enrichment analysis identified a marked downregulation of RNA splicing related genes in *BCLAF1*-low patients from both the adult BEAT-AML (fig. S3C) and the pediatric TARGET-AML (fig. S3D) cohorts, indicating a link between *BCLAF1* expression and splicing-related transcriptional programs in primary AML. Notably, analysis of the BEAT-AML cohort showed that *BCLAF1* expression is overall increased in cases harboring mutations in core splicing factors (fig. S3E), further supporting its association with splicing dysregulation in primary AML.

The association of BCLAF1 with the spliceosome in AML cells prompted us to investigate its potential role in regulating mRNA splicing. We evaluated changes in alternative splicing by RNA sequencing (RNA-seq) of MOLM-13 cells upon depletion of BCLAF1. Using the quantification tool MAJIQ (*44*), we found 1503 significantly altered splicing events (FDR ≤ 0.1, Δ percent spliced in (PSI) ≥ 0.05) (table S4). The most frequently affected category was intron retention, accounting for nearly half of the total events (Fig. 2, E and F). Isoform-specific RT-qPCR validated BCLAF1-dependent splicing alterations of selected targets (Fig. 2G), and similar results were obtained using a second shRNA targeting *BCLAF1* (fig. S3F). Altogether, these findings indicate that loss of BCLAF1 leads to widespread alterations in mRNA splicing.

To assess whether BCLAF1 directly associates with target mRNAs that undergo splicing changes, we performed BCLAF1 RNA immunoprecipitation sequencing (RIP-seq) experiments in MOLM-13 cells and identified 3567 significantly enriched transcripts in the BCLAF1-IP compared to input control (FDR ≤ 0.05, FC ≥ 1.5) (table S5). Gene ontology analysis of BCLAF1-bound transcripts showed an enrichment for pathways related to mitotic and DNA double-strand break repair processes (fig. S3G), consistent with previously described roles of BCLAF1 in maintaining genome stability and cell cycle progression (*39*, *40*, *45*). We also found an enrichment for mRNA splicing related categories (fig. S3G), reinforcing the notion that BCLAF1 contributes to the maintenance of the splicing machinery in AML cells. Interestingly, integration of the RIP-seq and RNA-seq datasets indicated a statistically significant overlap between transcripts bound by BCLAF1 and those exhibiting splicing alterations upon BCLAF1 knockdown (fig. S3H). This suggests that BCLAF1 controls alternative splicing, at least in part, through specific association with pre-mRNA targets. We validated a subset of these interactions by performing RIP-qPCR experiments, confirming both the specific association of BCLAF1 to selected transcripts and the loss of enrichment upon its depletion (Fig. 2H). Collectively, these results indicate that BCLAF1 regulates mRNA splicing in AML cells, which is consistent with its association with the spliceosome complex and localization to nuclear speckles.

### BCLAF1 promotes amino acid metabolic gene expression

Our findings are consistent with a model in which BCLAF1 supports leukemic cell fitness through modulation of mRNA splicing. To further dissect the molecular pathways underlying its role in AML, we performed differential gene expression analysis in MOLM-13 cells following depletion of BCLAF1. This analysis identified 539 differentially expressed genes (FDR ≤ 0.05, FC ≥ 2) between shBCLAF1 and shCONTROL cells, including 226 up-regulated and 313 down-regulated transcripts (fig. S4A and table S6).

Gene ontology analysis of up-regulated transcripts showed enrichment for immune-related processes such as leukocyte migration (GO:0050900) and cell activation involved in immune response (GO:0002263) (Fig. 3A), which would be consistent with a shift toward a less immature leukemic state. In line with this, analysis of established AML hierarchy signatures further showed reduced enrichment of primitive/progenitor-associated programs and increased enrichment of more mature myeloid-like programs (Fig. 3B). Moreover, we observed a marked downregulation of multiple *HOXA* cluster transcripts (fig. S4A), which are key regulators of leukemic transformation (*46*). RT-qPCR analysis confirmed reduced expression of selected *HOXA* mRNAs upon BCLAF1 depletion (Fig. 3C), and similar results were obtained using a second shRNA targeting BCLAF1 (fig. S4B).

**Fig. 3.**
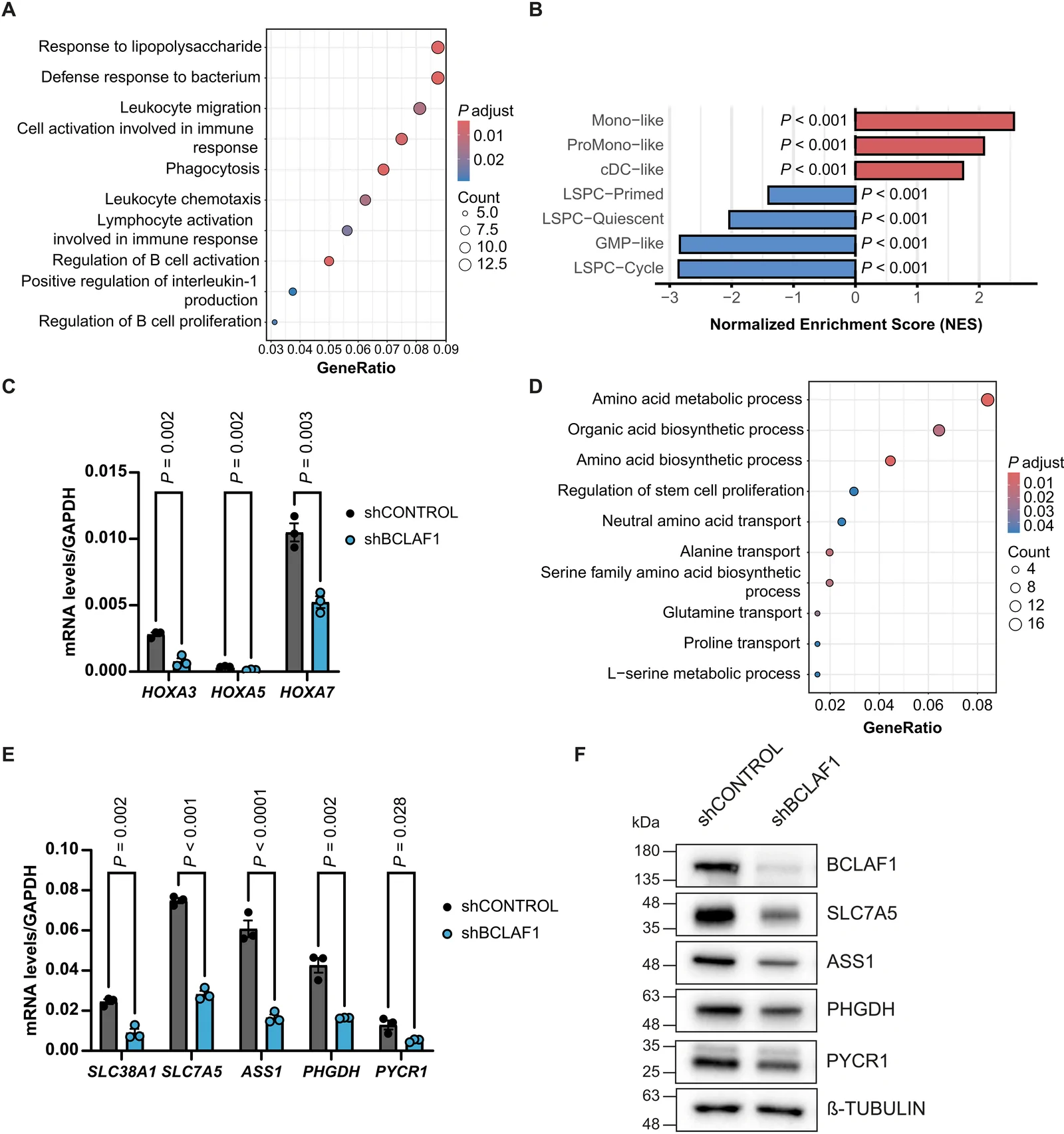
BCLAF1 promotes amino acid metabolic gene expression. (**A**) Dot plot of GO terms enriched among genes up-regulated upon BCLAF1 depletion in MOLM-13 cells. (**B**) Gene set enrichment analysis (GSEA) of AML hierarchy signatures in shBCLAF1 versus shCONTROL MOLM-13 cells. (**C**) RT-qPCR experiments showing relative mRNA levels of the indicated transcripts in shCONTROL and shBCLAF1 MOLM-13 cells treated with DOX for 96 hours. Data were normalized to *GAPDH* and represent mean ± SEM of three biological replicates. Statistics by unpaired *t* test. (**D**) Dot plot of GO terms enriched among genes down-regulated upon BCLAF1 depletion in MOLM-13 cells. (**E**) RT-qPCR experiments showing relative mRNA levels of the indicated transcripts in shCONTROL and shBCLAF1 MOLM-13 cells treated with DOX for 96 hours. Data were normalized to *GAPDH* and represent mean ± SEM of three biological replicates. Statistics by unpaired *t* test. (**F**) Immunoblot analysis of BCLAF1, SLC7A5, ASS1, PHGDH and PYCR1 protein levels in shCONTROL and shBCLAF1 MOLM-13 cells treated or not with DOX for 96 hours. β-tubulin was used as a loading control.

We next focused on the subset of transcripts that were down-regulated upon BCLAF1 depletion. Gene ontology analysis showed a marked enrichment for pathways related to amino acid metabolism, particularly transport and biosynthesis (Fig. 3D). These results were confirmed by RT-qPCR analysis, showing downregulation of representative genes associated with amino acid transport (*SLC38A1*, *SLC7A5*) and biosynthesis (*ASS1*, *PHGDH*, *PYCR1*) (Fig. 3E), and similar results were obtained using a second independent shRNA targeting BCLAF1 (fig. S4C). In line with these transcriptional changes, immunoblot analysis confirmed reduced steady-state protein levels of SLC7A5, ASS1, PHGDH and PYCR upon BCLAF1 depletion (Fig. 3F), further supporting the notion that BCLAF1 loss compromises amino acid metabolic capacity at both the transcript and protein levels.

Collectively, our findings establish the downregulation of amino acid metabolic genes as a downstream consequence of BCLAF1 loss. Given the crucial role of amino acid metabolism in supporting leukemic cell growth and survival (*15*–*18*, *47*), this may represent one mechanism through which BCLAF1 supports the undifferentiated leukemic state.

### Loss of BCLAF1 disrupts de novo amino acid synthesis

Based on our differential gene expression analysis, we next asked whether BCLAF1-dependent transcriptional changes translated into functional alterations in amino acid metabolism. We first assessed steady-state intracellular amino acid levels, which reflect the combined effects of synthesis, import, and catabolism. Gas chromatography–mass spectrometry (GC–MS) analysis revealed that while the total abundance of most detected amino acids was largely preserved upon BCLAF1 depletion, intracellular levels of proline and arginine were significantly reduced (fig. S5A and table S7), indicating selective perturbations in amino acid homeostasis.

To directly assess de novo amino acid synthesis, we performed isotope tracing experiments in glucose-free media supplemented with a glucose isotopologue containing six heavy carbons (U-13C-glucose) for 24 hours. Labeling of upstream glycolytic intermediates, including glyceraldehyde-3-phosphate and pyruvate, as well as TCA cycle intermediates such as α-ketoglutarate, citrate and succinate, was comparable between shCONTROL and shBCLAF1 cells (Fig. 4A and fig. S5, B and C), indicating that central carbon metabolism remained intact. Consistent with this, steady-state levels of TCA-derived amino acids, including aspartate and glutamate, were not significantly altered upon BCLAF1 depletion (fig. S5D). In contrast, BCLAF1 knockdown cells displayed a marked reduction in the incorporation of ^13^C from glucose into serine, as evidenced by a significantly lower proportion of labeled serine isotopologues (the sum of M + 1, M + 2 and M + 3, red) (4.29% vs. 15.45%, *P* < 0.05), accompanied by an accumulation of unlabeled serine (M + 0, grey) (Fig. 4B). This defect propagated downstream, resulting in significantly diminished labeling of glycine (2.57% vs. 7.2%, *P* < 0.01) and methionine (3.26% vs. 5.77%, p < 0.05) (Fig. 4, C and D). Despite preserved glycolytic flux, alanine labeling was also significantly reduced (68.01% vs. 87.65%, *P* < 0.001) (Fig. 4E), indicating that defects in de novo amino acid synthesis extend beyond the serine–glycine pathway. Consistent with these metabolic alterations, expression of key biosynthetic enzymes involved in serine production (PHGDH, PSAT1, and PSPH), serine-to-glycine conversion (SHMT2), and alanine synthesis (GPT2) was markedly reduced upon BCLAF1 knockdown (Fig. 4F), providing a direct mechanistic basis for the observed defects. Collectively, these findings suggest that BCLAF1 loss selectively disrupts de novo amino acid synthesis through diminished expression of its associated metabolic enzymes.

**Fig. 4.**
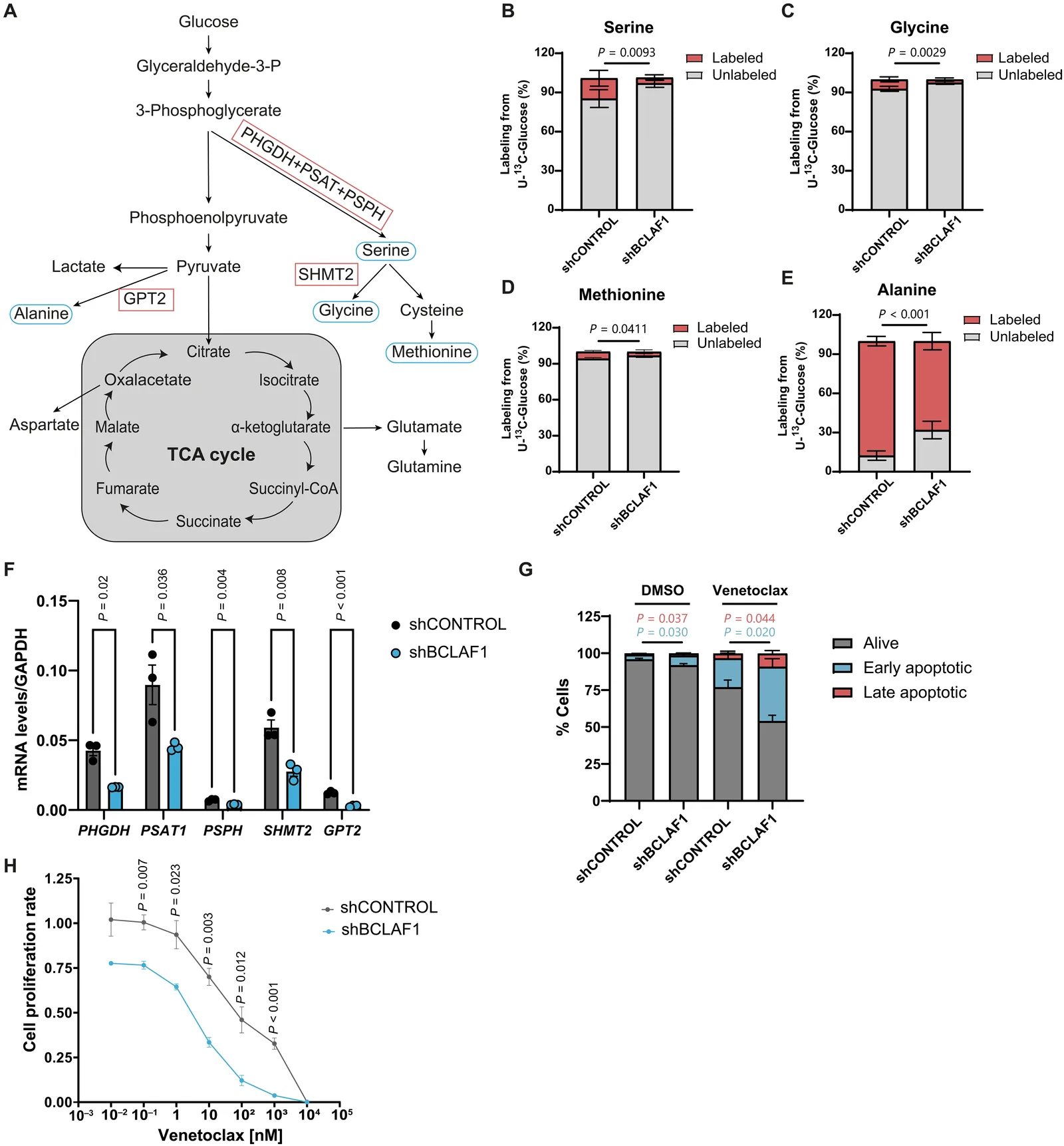
Loss of BCLAF1 disrupts de novo amino acid synthesis. (**A**) Schematic of amino acid biosynthesis pathways. (**B**), (**C**), (**D**) and (**E**) Percentage of unlabeled (M + 0; gray) or labeled (the sum of M + 1, M + 2, and M + 3; red) amino acid levels. Data are shown as mean ± SEM of four technical replicates from one representative experiment. Statistics by two-way ANOVA. The experiment was independently repeated with similar results. (**F**) RT-qPCR experiments showing relative mRNA levels of the indicated transcripts in shCONTROL and shBCLAF1 MOLM-13 cells treated with DOX for 96 hours. Data were normalized to *GAPDH* and represent mean ± SEM of three biological replicates. Statistics by unpaired *t* test. (**G**) annexin V/PI flow cytometry analysis showing the percentage of alive, early apoptotic and late apoptotic shCONTROL and shBCLAF1 MOLM-13 cells after 96 hours of DOX induction followed by 24 hour treatment with either DMSO or 50 nM venetoclax. Data represent mean ± SEM of three biological replicates. Statistics by unpaired *t* test (**H**) Cell viability assay (CellTiter-Blue-based) in shCONTROL and shBCLAF1 MOLM-13 cells treated with DOX for 96 hours, followed by 96 additional hours of Venetoclax treatment. Data is normalized to control (DMSO) and represent mean ± SEM of four biological replicates. Statistics by unpaired *t* test.

Next, we investigated whether exogenous amino acid supplementation could restore the growth defects observed upon BCLAF1 depletion. However, increasing concentrations of selected amino acid mixtures failed to rescue this proliferation defect (fig. S5E), suggesting that the metabolic consequences of BCLAF1 loss extend beyond impaired de novo amino acid synthesis. This is consistent with the reduced expression of amino acid transporters observed upon BCLAF1 depletion (Fig. 3, D, E, and F) and points to a broader disruption of amino acid homeostasis involving both biosynthesis and transport.

Amino acid metabolism plays a central role in AML maintenance (*48*). Therefore, we asked whether BCLAF1 depletion could influence the cellular response to venetoclax, a BCL-2 inhibitor shown to impair amino acid uptake and mitochondrial metabolism in LSCs (*15*). Notably, loss of BCLAF1 enhanced venetoclax-induced apoptosis compared to control cells, as assessed by annexin V/PI staining (Fig. 4G). Consistently, dose-response viability assays showed that BCLAF1-depleted cells were more sensitive to venetoclax (Fig. 4H). Together, these findings indicate that BCLAF1 loss increases susceptibility to venetoclax-induced cell death, suggesting a potential therapeutic vulnerability in AML.

### BCLAF1 supports amino acid metabolism through ATF4

Our transcriptomic analyses in MOLM-13 cells indicated that depletion of BCLAF1 leads to widespread changes in both gene expression and mRNA splicing. However, integration of differential expression and alternative splicing analyses revealed limited overlap (fig. S6A), suggesting that most BCLAF1-dependent splicing alterations do not directly impact global steady-state mRNA abundance in AML cells.

To better understand how BCLAF1 depletion drives transcriptional changes, we performed an unbiased transcription factor enrichment analysis using the TRRUST database (*49*). This analysis identified ATF4, a key transcriptional regulator of amino acid metabolism (*50*), as the most significantly enriched factor among the BCLAF1-downregulated gene set (table S8). Supporting this connection, analysis of ENCODE ChIP-seq data for ATF4 in K562 AML cells revealed that 75% (18 of 24) of BCLAF1-downregulated genes annotated in amino-acid metabolic pathways display ATF4 binding (fig. S5, B and C). These data suggest that the transcriptional effects of BCLAF1 depletion may be mediated, at least in part, through reduced ATF4 activity. In line with this hypothesis, immunoblot analysis of MOLM-13 cells showed a marked reduction in ATF4 protein levels upon BCLAF1 depletion (Fig. 5A). Likewise, MLL-AF9 leukemias derived from *Bclaf1*-deficient *Vav-Cre:Bclaf1^f/f^* mice exhibited decreased ATF4 protein levels compared to leukemias derived from wild-type *Bclaf1^f/f^* controls (Fig. 5B), suggesting that BCLAF1 supports ATF4 expression in primary AML cells.

**Fig. 5.**
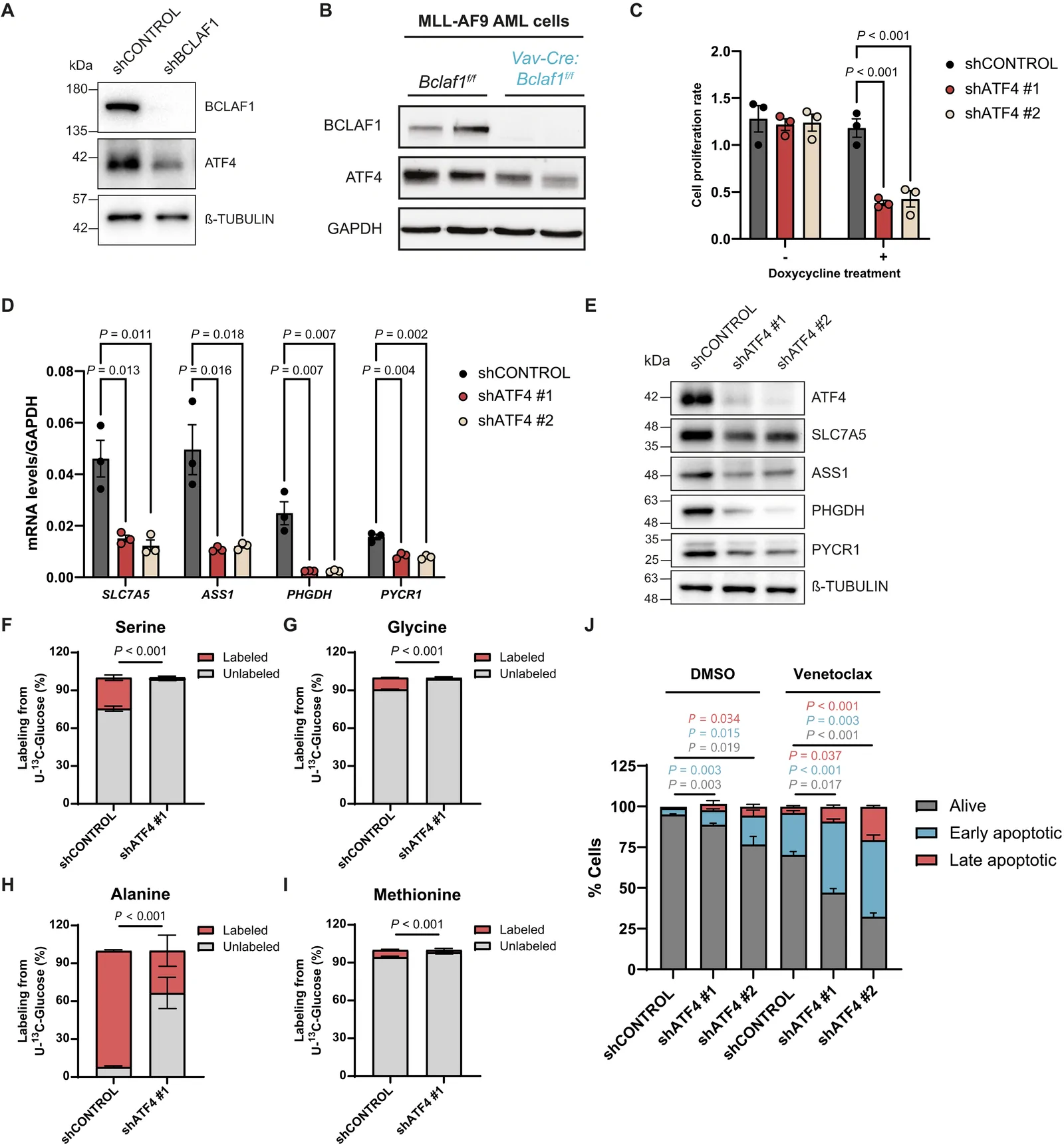
BCLAF1 supports amino acid metabolism through ATF4. (**A**) Immunoblot analysis of BCLAF1 and ATF4 protein levels in shCONTROL and shBCLAF1 MOLM-13 cells treated with DOX for 96 hours. β-tubulin was used as a loading control. (**B**) Immunoblot analysis of BCLAF1 and ATF4 protein levels in MLL-AF9-expressing murine AML cells of indicated genotypes. GAPDH was used as a loading control. (**C**) Cell proliferation assay (CellTiter-Blue-based) in shCONTROL and shATF4 #1 and #2 MOLM-13 cells treated or not with DOX for 96 hours, followed by 96 additional hours in culture. Data represent mean ± SEM of three biological replicates. Statistics by unpaired *t* test. (**D**) RT-qPCR experiments showing relative mRNA levels of the indicated transcripts in shCONTROL and shATF4 #1 and #2 MOLM-13 cells treated with DOX for 96 hours. Data were normalized to *GAPDH* and represent mean ± SEM of three biological replicates. Statistics by unpaired *t* test. (**E**) Immunoblot analysis of ATF4, SLC7A5, ASS1, PHGDH and PYCR1 protein levels in shCONTROL and shATF4 #1 and #2 MOLM-13 cells treated with DOX for 96 hours. β-tubulin was used as a loading control. (**F**), (**G**), (**H**) and (**I**) Percentage of unlabeled (M + 0; gray) and labeled (sum of M + 1, M + 2 and M + 3; red) amino acid isotopologues. Data represent mean ± SEM of four technical replicates. Statistics by two-way ANOVA. (**J**) Annexin V/PI flow cytometry analysis showing the percentage of alive, early apoptotic and late apoptotic shCONTROL and shATF4 #1/#2 MOLM-13 cells after 96 hours of DOX induction followed by 24 hour treatment with either DMSO or 50 nM venetoclax. Data represent mean ± SEM of three biological replicates. Statistics by unpaired *t* test.

To determine whether this relationship between BCLAF1 and ATF4 expression levels extends beyond MLL-AF9-driven leukemias, we examined OCI-AML3 cells, which carry *NPM1* and *DNMT3A* mutations, and HL-60 cells, which carry *MYC* amplification and *NRAS* mutation. In both cell lines, BCLAF1 depletion also led to a pronounced reduction in ATF4 protein levels (fig. S6D), which was accompanied by downregulation of ATF4 target genes involved in amino acid metabolism (fig. S6, E and F), impaired cell proliferation (fig. S6, G and H) and increased sensitivity to venetoclax treatment (fig. S6, I and J). These data indicate that the BCLAF1–ATF4 regulatory axis operates across genetically distinct AML subtypes.

ATF4 activity and target gene expression are known to be elevated in primary LSCs (*51*), and several studies have implicated ATF4 in AML pathogenesis (*52*–*54*). However, the precise molecular contribution of ATF4 to AML maintenance remains incompletely defined. To determine whether ATF4 contributes to the functional effects of BCLAF1 loss, we generated MOLM-13 cell lines expressing two independent DOX-inducible shRNAs targeting *ATF4*. ATF4 depletion did not alter BCLAF1 protein levels (fig. S6K), consistent with BCLAF1 functioning upstream of ATF4. Notably, ATF4 knockdown phenocopied BCLAF1 depletion, resulting in impaired cell proliferation (Fig. 5C) and reduced expression of amino acid metabolic genes at both the mRNA (Fig. 5D) and protein (Fig. 5E) levels. Moreover, loss of ATF4 similarly impaired de novo amino acid synthesis (Fig. 5, F, G, H, and I and table S9) and enhanced venetoclax-induced apoptosis compared with control cells (Fig. 5J). Taken together, these findings support a model in which BCLAF1 promotes amino acid metabolic programs important for AML maintenance through regulation of ATF4 expression.

### BCLAF1 sustains ATF4 expression independently of canonical ISR activation

ATF4 protein expression is controlled by the Integrated Stress Response (ISR), a conserved adaptive pathway that promotes its selective translation upon phosphorylation of eukaryotic translation initiation factor 2A (eIF2α) (*55*). The ISR is activated by four main stress-responsive kinases (PERK, GCN2, PKR, and HRI), which converge on eIF2α phosphorylation to attenuate global translation while selectively enhancing ATF4 synthesis. Emerging evidence suggests that ISR signaling contributes to LSC maintenance and leukemogenesis (*51*, *56*). Therefore, we investigated whether BCLAF1 regulates ATF4 expression through modulation of ISR activity.

To examine ISR status, we treated cells with tunicamycin, a potent inducer of endoplasmic reticulum stress and PERK-mediated ISR activation. Under basal conditions, levels of ISR kinases and eIF2α phosphorylation remained stable upon BCLAF1 depletion (Fig. 6A). Tunicamycin treatment induced robust phosphorylation of PERK and eIF2α in both control and BCLAF1-depleted cells (Fig. 6A), indicating that upstream ISR activation is largely preserved. In line with this, loss of BCLAF1 did not alter cellular sensitivity to tunicamycin (Fig. 6B). Notably, ATF4 protein levels remained lower in BCLAF1-depleted cells regardless of tunicamycin treatment (Fig. 6A), suggesting that reduced ATF4 expression cannot be explained by defective canonical ISR activation.

**Fig. 6.**
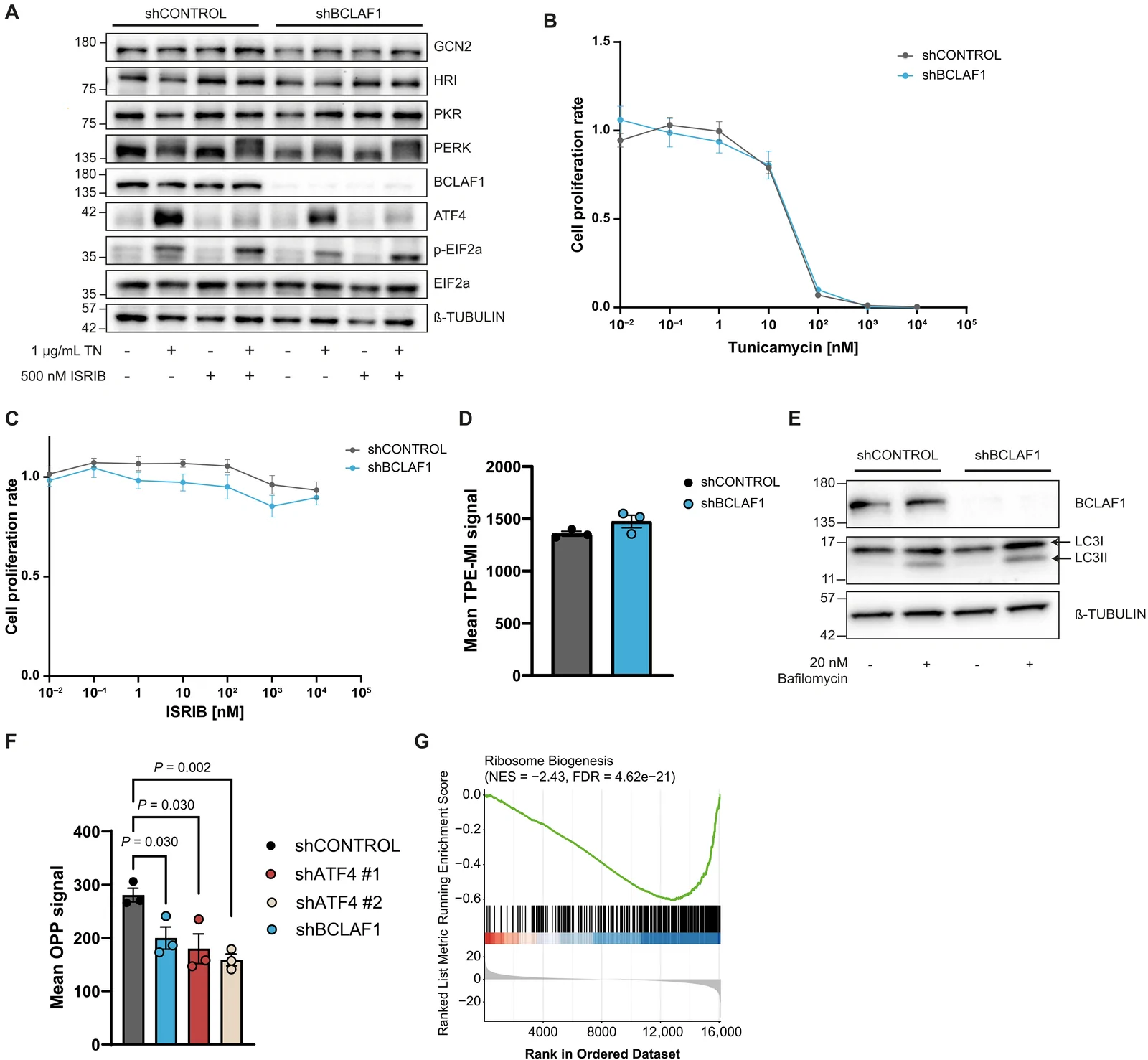
BCLAF1 sustains ATF4 expression independently of canonical ISR activation. (**A**) Immunoblot analysis of GCN2, HRI, PKR, PERK, BCLAF1, ATF4, p-eIF2α and total eIF2α protein levels in shCONTROL and shBCLAF1 MOLM-13 cells treated with DOX for 96 hours, followed by 3 hour treatment with 1 μg/mL tunicamycin and/or 500 nM ISRIB. Untreated cells were incubated with DMSO. β-tubulin was used as a loading control. (**B**) CellTiter-Blue viability assay in shCONTROL and shBCLAF1 MOLM-13 cells treated with DOX for 96 hours, followed by an additional 96 hours of tunicamycin treatment. Data were normalized to the corresponding DMSO control and represent mean ± SEM of four biological replicates. Statistics by unpaired *t* test. (**C**) CellTiter-Blue viability assay in shCONTROL and shBCLAF1 MOLM-13 cells treated with DOX for 96 hours, followed by an additional 96 hours of ISRIB treatment. Data were normalized to the corresponding DMSO control and represent mean ± SEM of four biological replicates. Statistics by unpaired *t* test. (**D**) Flow cytometry analysis of unfolded protein burden in shCONTROL and shBCLAF1 MOLM-13 cells after 96 hours of DOX induction, measured using TPE-MI mean fluorescence intensity. Data represent mean ± SEM of three biological replicates. Statistics by unpaired *t* test. (**E**) Immunoblot analysis of BCLAF1 and LC3I/II protein levels in shCONTROL and shBCLAF1 MOLM-13 cells treated with DOX for 96 hours, followed by 2 hour treatment with either 20 nM bafilomycin or DMSO. β-tubulin was used as a loading control. (**F**) Flow cytometry analysis of O-propargyl-puromycin (OPP) incorporation assays in shCONTROL, shBCLAF1 and shATF4 #1 and #2 MOLM-13 cells. Data represent mean ± SEM of three biological replicates. Statistics by unpaired *t* test. (**G**) Gene set enrichment analysis (GSEA) of ribosome biogenesis-related genes in shBCLAF1 versus shCONTROL MOLM-13 cells.

To functionally assess ISR dependence, we treated shCONTROL and shBCLAF1 cells with ISRIB, a small molecule that counteracts the effects of eIF2α phosphorylation (*57*). ISRIB treatment had minimal impact on the proliferation of control and BCLAF1-depleted cells across all concentrations tested (Fig. 6C), further supporting the notion that BCLAF1 loss does not impair canonical ISR signaling. Consistent with this, BCLAF1 depletion did not induce proteostasis stress (Fig. 6D) or compensatory autophagy activation (Fig. 6E), which have been recently implicated in regulating ISR initiation thresholds in AML cells (*58*).

Despite the absence of increased eIF2α phosphorylation, O-propargyl-puromycin (OPP) incorporation assays showed a reduction in nascent protein synthesis following BCLAF1 depletion (Fig. 6F). This observation was unexpected and raises the possibility that BCLAF1 influences translational output through an alternative mechanism. It has been recently reported that ATF4 modulates ribosome biogenesis during erythropoiesis (*59*), raising the possibility that reduced translational output in BCLAF1-depleted cells arises as a downstream consequence of impaired ATF4 function. In line with this, ATF4 knockdown phenocopied BCLAF1 depletion (Fig. 6F), resulting in reduced OPP incorporation. Moreover, gene set enrichment analysis of MOLM-13 RNA-seq data identified a marked downregulation of ribosome biogenesis-related genes upon depletion of BCLAF1 (Fig. 6G). Taken together, these findings indicate that BCLAF1 sustains ATF4 expression and downstream translational capacity independently of canonical ISR activation in AML cells.

### BCLAF1 controls *ATF*4 mRNA alternative splicing

Next, we sought to determine how BCLAF1 regulates ATF4 expression. *ATF4* mRNA has two major splice variants: variant 1 (V1, ENST00000337304.2), which retains the first intron, and variant 2 (V2, ENST00000674920.3), which encodes the canonical ATF4 protein (fig. S7A). V1 is highly unstable in AML cells due to its nuclear retention and degradation, whereas V2 is efficiently exported and translated into functional ATF4 protein (*60*). To investigate whether BCLAF1 regulates this splicing event, we performed isoform-specific RT-qPCR analysis. We first confirmed that V1 is expressed at substantially lower levels than V2 under basal conditions (fig. S7B), as previously reported (*60*). Upon BCLAF1 depletion, we observed a notable increase in the V1/V2 ratio (Fig. 7A), indicating a shift in splicing toward the intron-retaining isoform. This shift reflected both an increase in V1 levels and a concomitant reduction in V2 (fig. S7C), suggesting that BCLAF1 controls the balance between these isoforms. As a result, total *ATF4* mRNA levels were reduced in BCLAF1-depleted cells (Fig. 7B), in line with the reported instability of the V1 isoform in AML cells (*60*). These results indicate that BCLAF1 promotes the productive splicing of *ATF4* pre-mRNA to the stable, protein-coding V2 isoform, thereby sustaining *ATF4* transcript abundance.

**Fig. 7.**
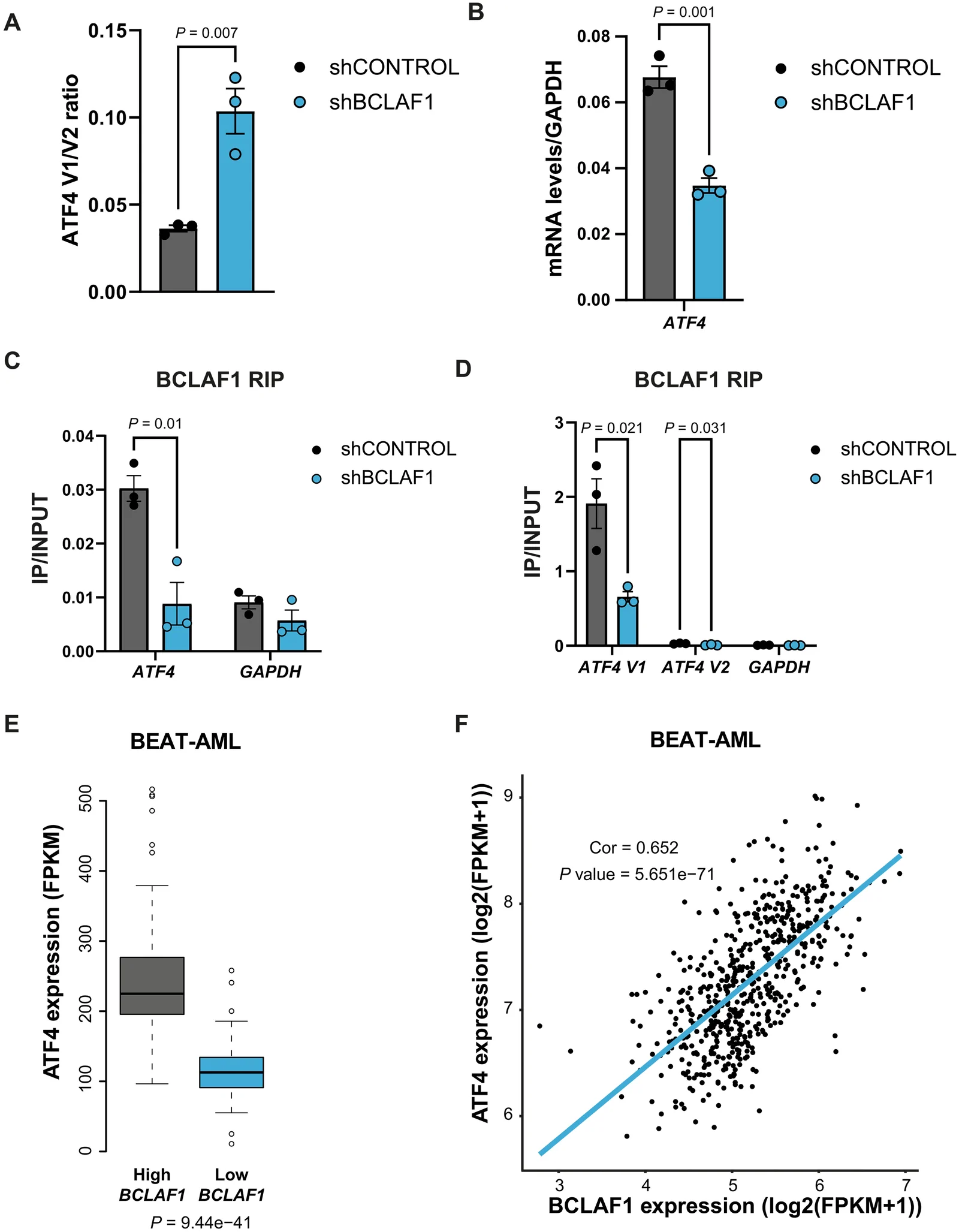
BCLAF1 controls *ATF4* mRNA alternative splicing. (**A**) and (**B**) RT-qPCR experiments showing relative mRNA levels of the indicated transcripts in shCONTROL and shBCLAF1 MOLM-13 cells treated with DOX for 96 hours. Data were normalized to *GAPDH* and represent mean ± SEM of three biological replicates. Statistics by unpaired *t* test. (**C**) and (**D**) RIP-qPCR experiments showing BCLAF1 association with the indicated transcripts in shCONTROL and shBCLAF1 MOLM-13 cells treated with DOX for 96 hours. *GAPDH* was used as a negative control. Data represent mean ± SEM of three biological replicates. Statistics by unpaired *t*test. (**E**) Box plot showing *ATF4* mRNA levels in *BCLAF1*-high (top 25%) and *BCLAF1*-low (bottom 25%) patient samples from the BEAT-AML cohort. Statistical significance was assessed using Wilcoxon Rank Sum and Signed Rank Tests (one-sided). (**F**) Scatter plot showing the correlation between *BCLAF1* and *ATF4* mRNA levels across BEAT-AML patient samples. Pearson correlation coefficient (*r*) and *P* value are indicated.

Our RIP-seq analysis identified *ATF*4 mRNA as a prominent BCLAF1-bound transcript (table S5). To validate this interaction, we performed RIP-qPCR experiments following BCLAF1 knockdown. We found that BCLAF1 specifically associates with the *ATF4* transcript, and that this interaction is reduced upon BCLAF1 depletion (Fig. 7C). Moreover, this interaction was markedly enriched for the V1 isoform when compared with V2 (Fig. 7D), suggesting preferential association of BCLAF1 with the unspliced pre-mRNA. This observation aligns with the presence of a conserved non-canonical weak splice donor site in intron 1 (*60*), and raises the possibility that BCLAF1 facilitates efficient splice site recognition, thereby promoting intron removal. Collectively, our data support a model in which BCLAF1 safeguards productive *ATF4* mRNA splicing by directly associating with its transcript. This regulatory step is required to sustain ATF4 protein levels and downstream transcriptional programs controlling amino acid metabolism in AML cells.

To validate the clinical relevance of these findings, we next analyzed RNA-seq data from the BEAT-AML cohort. Notably, we found that *BCLAF1*-low patients displayed significantly reduced *ATF4* mRNA levels compared to *BCLAF1*-high cases (Fig. 7E). Consistent with this, plotting *BCLAF1* versus *ATF4* mRNA levels revealed a strong positive correlation across all patient samples (Fig. 7F), indicating that this regulatory relationship extends to primary human AML. This association was also observed, although to a lesser extent, in the pediatric TARGET-AML cohort (fig. S7, D and E), further reinforcing the potential clinical relevance of the BCLAF1–ATF4 axis. Taken together, these data indicate that BCLAF1 expression is closely linked to ATF4 levels in primary AML.

## DISCUSSION

Multiple studies have identified distinct RNA-binding proteins (RBPs) as essential for leukemic cell survival (*29*, *61*–*65*), underscoring the importance of post-transcriptional control in AML pathogenesis. Alternative mRNA splicing has been shown to alter the expression of key AML-associated genes independently of somatic mutations, thereby contributing to leukemogenesis and disease progression (*66*, *67*). Moreover, dysregulation of splicing regulatory mechanisms has been implicated in therapy resistance (*68*). Together, these findings position splicing regulation as a key contributor to AML evolution and therapeutic response. In this study, we identify BCLAF1 as an important post-transcriptional regulator in AML and show that it promotes leukemic maintenance by regulating alternative mRNA splicing, establishing it as a fundamental RBP dependency in AML.

BCLAF1 was originally identified as a BCL2-interacting protein with pro-apoptotic and transcriptional repressor functions (*69*). However, subsequent studies have revealed broader and context-dependent roles, including functions in DNA damage response, viral infection, and cellular differentiation (*70*). More recently, BCLAF1 has emerged as an RNA-binding protein involved in mRNA metabolism (*39*, *40*, *45*, *71*, *72*). Here, we identify a role for BCLAF1 in regulating mRNA splicing programs in AML, distinct from its originally described association with BCL2. We show that BCLAF1 physically interacts with core spliceosome components and regulates hundreds of alternative splicing events in AML cells, with a predominant impact on intron retention. Our data indicate that these effects are mediated, at least in part, through its specific association with mRNA targets, supporting a model in which BCLAF1 functions as a splicing co-regulator. Consistent with this, we found that BCLAF1 expression is elevated in AML patients harboring mutations in core splicing factor genes, suggesting that its function may be particularly relevant in this genetic context. Whether elevated BCLAF1 expression contributes to adaptive metabolic responses previously observed in spliceosome-mutant myeloid malignancies remains to be determined (*25*–*27*). Overall, these findings provide mechanistic insight into previous observations linking BCLAF1 to AML pathogenesis (*73*–*75*) and further establish it as a central post-transcriptional regulator in leukemia.

We identify the stress-responsive transcription factor ATF4, a fundamental regulator of amino acid metabolism and cellular homeostasis (*50*, *76*), as a key downstream effector of BCLAF1. BCLAF1 associates with *ATF4* mRNA and regulates its splicing to minimize an intron-retaining, unstable variant and maintain a stable, protein coding isoform. Both ATF4 activity and target gene expression have been shown to be elevated in primary LSCs (*51*), and multiple studies have implicated ATF4 in supporting AML pathogenesis (*52*–*54*). Our findings position BCLAF1 as an upstream post-transcriptional regulator of ATF4, uncovering a mechanistic link by which splicing control contributes to amino acid metabolic adaptation and leukemic maintenance.

Recent studies have highlighted the therapeutic potential of targeting amino acid metabolic pathways in AML (*14*, *48*). For example, reducing amino acid import through the combination of venetoclax and azacitidine selectively eliminates LSCs by impairing mitochondrial respiration (*15*). Moreover, AML cells display distinct metabolic dependencies on specific amino acids, including on glutamine (*17*, *47*), serine (*18*, *21*) and arginine (*19*, *20*). However, leukemia cells can adapt to nutrient stress by inducing the expression of ATF4 (*77*), which could explain the limited efficacy of amino acid deprivation strategies when used as monotherapies (*78*, *79*). Our results suggest that BCLAF1 may contribute to this adaptive capacity by regulating ATF4 expression. We show that BCLAF1 depletion enhances venetoclax-induced apoptosis and that reduced ATF4 activity likely contributes to this phenotype. Consistent with this, a recent study demonstrated that an ATF4-dependent serine metabolic program contributes to venetoclax resistance in AML (*80*). Together, these observations suggest that the BCLAF1–ATF4 axis may influence AML response to venetoclax, although the mechanistic basis and therapeutic relevance of this association remain to be defined.

BCLAF1 is ubiquitously expressed but displays cell-type dependent functions (*70*). During tumorigenesis, it can act as either a tumor promoter (*36*, *37*, *81*–*84*) or tumor suppressor (*85*–*88*) depending on the cancer type and cellular context. Interestingly, our previous work showed that although BCLAF1 promotes fetal hematopoietic stem cell (HSC) development and hematopoietic reconstitution after transplantation, it is dispensable for the maintenance of adult HSCs under steady-state conditions (*28*). In that study, deletion of BCLAF1 in adult HSCs had no effect on their numbers at steady state and did not impair hematopoietic recovery after chemotherapy or inflammatory stress. This differential requirement raises the possibility that targeting BCLAF1 may preferentially impair leukemic cells relative to normal hematopoiesis. Further studies will be required to define the extent and limits of this potential therapeutic window in AML.

## MATERIALS AND METHODS

### Cell lines

Human tumor cell lines MOLM-13 (male; DSMZ, ACC-554; RRID CVCL_2119), THP-1 (male; ATCC, TIB-202; RRID, CVCL_0006) and HL-60 (DSMZ, ACC 3; RRID, CVCL_0002) cells were cultured in RPMI 1640-Glutamax (61870036, GIBCO) supplemented with 10% FBS (16SV30160.03, CYTIVA) and 1% penicillin/streptomycin (5525–053-Cl, CYTIVA). OCI-AML3 (male; DSMZ, ACC 582; CVCL_1844) cells were cultured in MEM alpha modified with L-glutamine, Ribo- y Deoxyribonucleosides, (SH30265.01, CYTIVA) supplemented with 10% FBS (16SV30160.03, CYTIVA) and 1% penicillin/streptomycin (5525–053-Cl, CYTIVA). HEK293T cells (female; ATCC, CRL-3216; RRID: CVCL_0063) were cultured in DMEM High Glucose (D6546, Sigma-Aldrich) supplemented with 10% FBS (16SV30160.03, CYTIVA) and 1% penicillin/streptomycin/glutamine (5525–053-Cl, 5525–005-Cl, CYTIVA). All cells were grown at 37°C in a humidified incubator with 5% CO_2_. MOLM-13, THP-1, OCI-AML3 and HL60 cells were used as AML models; HEK293T for lentiviral production. Cell lines were regularly tested and confirmed negative for mycoplasma.

### Mice

*Bclaf1f/f* mice (RRID:MMRRC_046783-UCD) were previously described and were obtained from the Mutant Mouse Resource and Research Center (MMRRC) at University of California at Davis, an NIH-funded strain repository, and were donated to the MMRRC by The KOMP Repository, University of California, Davis. Vav-Cre mice (RRID:IMSR_JAX:008610) and Mx-Cre mice (RRID:IMSR_JAX:003556) were purchased from The Jackson Laboratory. CD45.1 mice (RRID:IMSR_CRL:494) mice were purchased from Charles River Laboratories. Mice were bred and housed according to institutional animal care and use committee standards at Washington University in St. Louis School of Medicine (IACUC approval no. 25–0159). Both sexes were used equivalently in all experiments.

#### Plasmids

pMSCV-IRES-GFP II (pMIG II) was a gift from Dario Vignali (Addgene plasmid #52107; RRID:Addgene_52107). pMIG-FLAG-*MLL-AF9* was a gift from Daisuke Nakada (Addgene plasmid #71443; RRID:Addgene_71443). Cre was cloned into pMSCV-IRES-Thy1.1 to create pMIT-Cre. pMSCV-IRES-Thy1.1 was a gift from Anjana Rao (Addgene plasmid #17442; RRID:Addgene_17442). Tet-pLKO-puro was a gift from Dmitri Wiederschain (Addgene plasmid # 21915; RRID:Addgene_21915), LentiCRISPRv2GFP was a gift from David Feldser (Addgene plasmid # 82416; RRID:Addgene_82416) and pHIV-Zsgreen was a gift from Bryan Welm & Zena Werb (Addgene plasmid # 18121; RRID:Addgene_18121). pVSV-G was a gift from Akitsu Hotta (Addgene plasmid # 138479; RRID:Addgene_138479). p8.91 was a gift from Simon Davis (Addgene plasmid # 187441; RRID:Addgene_187441).

### Virus production

For lentiviral production HEK293T cells were transfected with the appropriate lentiviral vector mix (PLKO.1 for shRNA, LentiCRISPR-v2GFP for CRISPR-Cas9 and pHIV-Zsgreen for overexpression) together with the packaging plasmids p8.91 and pVSVg at a 1:1.5:0.5 using Lipofectamine 2000 reagent (Thermo Fisher Scientific, 11668019). Specific oligos for each plasmid are indicated in table S10. Supernatant was harvested 48 and 72 hours after transfection. For human cells transduction, cells and viral supernatants were mixed with 8 μg/ml (human) polybrene (TR-1003-G, Millipore), followed by spinfection (90 min, 850 *g*, RT) and further incubated overnight at 37°C. Cells were collected on the following day. Retrovirus was produced using Phoenix Eco cells (ATCC Cat# CRL-3214, RRID:CVCL_H717). On day 1, cells were plated at 500,000 cells/well in a 6-well plate in DMEM media containing MEM non-essential amino acids, glutamine, sodium pyruvate, beta-mercaptoethanol, penicillin, streptomycin, and 10% fetal bovine serum. On day 2, cell media was changed to antibiotic free media. Plasmid DNA was mixed and incubated with lipofectamine (Invitrogen) before dropwise transfection according to manufacturer’s protocol. On day 3, lipofectamine-containing media was replaced with standard media. On day 4, virus-containing media was harvested from the supernatant of cells.

### MLL-AF9 leukemia studies

CD45.2^+^ *Bclaf1f/f* or *Vav-Cre:Bclaf1f/f* donor mice were injected intraperitoneally with 150 mg/kg 5-fluorouracil (5-FU) 6 days prior to cell collection. 5-FU (Sigma #F6627) was dissolved to 25 mg/ml in PBS and pH was adjusted to 9–10 with 10 N NaOH. On day 6 (after 5-FU), bone marrow was collected from hind limbs, spine, and pelvis then enriched for c-Kit (CD117)-expressing cells using magnetic beads. Briefly, cells were incubated with biotinylated anti-c-Kit antibody (Biolegend #105803) for 20 minutes, then washed and incubated with MojoSort Streptavidin nanobeads (Biolegend #480016) for 15 minutes. Cells were washed then passed through magnetic MS columns (Miltenyi #130–042-201) according to manufacturer’s instructions. Enriched cells were resuspended at 2 × 10^6^ cells/mL in transduction media (Stemspan SFEM (StemCell Technologies), 100 ng/ml recombinant murine TPO, 100 ng/mL recombinant murine SCF (PeproTech), penicillin/streptomycin (Gibco)) and plated with polybrene (5 mg/mL, Sigma) and equivalent volumen (1:1) of viral supernatant in a 48-well plate. Cells were spinfected for 2 hours at room temperature at 1600 rpm. After spinfection, media was replaced with fresh transduction media with no polybrene, and cells were cultured overnight. The following morning, cells were spinfected again (as above) and cultured with fresh media for 6 hours before harvest and transplantation. For all transplants, CD45.1^+^ recipient mice received a split dose (≥3 hours apart) of lethal irradiation (11 Gy total). Primary recipients were administered 50,000–100,000 infected CD45.2^+^ donor cells with 300,000 CD45.1^+^ support cells. Cells were administered via retroorbital injection, and recipients were treated with trimethoprim/sulfamethoxazole for 14 days posttransplant. Mice were monitored weekly by peripheral blood GFP^+^ analysis and euthanized when peripheral blood showed ≥80% GFP positivity. Bone marrow and spleen were analyzed by flow cytometry for CD45.2^+^GFP^+^ blasts.

### In vitro murine AML studies

*Bclaf1f/f* MLL-AF9 cells harvested from transplanted mice as above were transduced with either pMSCV-IRES-Thy1.1 or pMCSV-IRES-Cre-Thy1.1 at 1500 rpm for 90 min, then cultured overnight in DMEM with 20% FBS and 10 ng/mL IL-3. Thy1.1^+^ cells were quantified by flow cytometry on day 7. For MIT-Cre CFU assays, Thy1.1^+^ sorted cells were plated in Methocult M3434 supplemented with IL-3. Colonies were counted at day 7.

### GFP-KO competition assay

GFP levels were measured by flow cytometry 2 days after infecting to set the starting baseline GFP percentage for each gRNA. The represented ratio was calculated using GFP^+^ cells day15 / GFP+ cells day2, which stands for the depletion of cells targeted for each guide. Two gRNAs were used to target BCLAF1, one for the Rosa26 locus as a negative control and one for RBM39 as a positive control. Flow cytometry data was analyzed using FlowJo software.

### CRISPR-Cas9 knockout of primary AML cells

Primary AML cells were obtained from deidentified banked samples under IRB 201011766, which was approved by the institutional review board at Washington University in St. Louis School of Medicine. All participants signed informed consent prior to collection of samples. Cells were cultured overnight in IMDM (Gibco) supplemented with 15% FBS (Sigma), 1% penicillin/streptomycin (Gibco), 1% Glutamax (Gibco), containing 10 ng/ml human IL-3 (Peprotech), 10 ng/ml human FLT3L (Peprotech), 100 ng/ml human SCF (Peprotech), 20 ng/ml human IL-6 (Peprotech), and 10 ng/ml human TPO (Peprotech).

The next day, 5-10x105 cells AML cells were used in each electroporation reaction. Ribonucleoprotein (RNP) complexes were formed by combining 3 μL of 61 μM recombinant Cas9 protein (IDT) with 2.1 μL of 100 μM sgRNA (BCLAF1 guides: AA [ataaggtcgtctcattcctc], #5 [ctgtacccataaggtcgtct], or HPRT negative control guide [tcctagtaatccccataatt]) in 14.9 μL of phosphate-buffered saline (PBS) for total volume of 20 μL. The mixture was incubated at room temperature for 15 min to facilitate stable RNP assembly. Cells were resuspended in 80 μL of P3 Nucleofector solution (Lonza) and combined with the RNP mixture plus 1.8 μL of Alt-R Cas9 Electroporation Enhancer (IDT). Electroporation was executed using the preset “human CD34+ HSPC” program on a 4D-Nucleofector system (Lonza). Immediately following pulse delivery, 180 μL of pre-warmed culture medium was added directly to the chamber. The entire volume was subsequently transferred to a 48-well plate supplemented with an additional 150 μL of culture medium per well and cultured overnight to allow cellular recovery.

Following overnight recovery, cells were plated in MethoCult H4435 Enriched media (Stem Cell technologies) at a concentration of 100,000 cells/ml. Colonies were manually counted 14 days later, and cells were harvested and counted. For genotyping analysis, DNA underwent amplicon expansion for target regions and sequencing was performed on a MiSeq i100 with 150 bp PE reads. A minimum of 60,000 reads was acquired per sample.

### Flow Cytometry

MOLM-13 and THP-1 inducible knockdown cell lines expressing shCONTROL or shBCLAF1 were collected after 4 days of doxycycline treatment. Cells were stained for surface CD11b expression or apoptosis using an annexin V-FITC kit (Miltenyi Biotec), according to the manufacturer’s instructions. Data were acquired on a FACSCalibur flow cytometer and analyzed using FlowJo. Mouse samples were stained for 20 min in staining buffer consisting of PBS supplemented with 2% FBS. The following antibodies were used: CD11b-APC, CD19-PE-Cy7, GR1-PerCP-Cy5.5, CD3-PE, CD45.1-APC-Cy7, CD45.2-AF700 and Thy1.1-APC. Cells were washed and resuspended in staining buffer containing DAPI to exclude dead cells. Flow cytometric analyses and sorting were performed on a BD LSRFortessa or BD FACSAria Fusion.

### O-propargyl-puromycin (OPP) incorporation assays

shCONTROL and shBCLAF1 MOLM-13 cells were treated with doxycycline for 96 hours and then incubated with 50 μM O-propargyl-puromycin (OPP; HY-15680, MedChemExpress) for 1 hour. Cells were washed once with PBS and fixed in methanol. To assess OPP incorporation, cells were permeabilized with 0.2% Triton X-100 for 10 min at room temperature, followed by two washes with PBS containing 1% BSA. Cells were then resuspended in Click-iT reaction buffer containing 100 mM Tris-HCl pH 8.8, 1 mM CuSO_4_, 100 mM L-ascorbic acid (A5960, Sigma) and 1 μM Alexa Fluor 647-azide (A10277, Thermo Fisher Scientific), and incubated in the dark for 30 min at room temperature. Cells were washed twice with PBS and analyzed by flow cytometry using a FACSCalibur and FlowJo.

### Tetraphenylethene maleimide fluorescence (TPE-MI) assay

After 4 days of doxycycline treatment, shCONTROL and shBCLAF1 MOLM-13 cells were washed twice with PBS. TPE-MI (HY-143218, MedChemExpress) was diluted in PBS to a final concentration of 50 μM, and cells were incubated for 45 min at 37°C protected from light. Samples were then washed twice with PBS and analyzed by flow cytometry using a BD LSRFortessa.

### Western blotting

Resolved proteins were transferred to either 0.45 μm PVDF membranes (IPVH00010, Millipore) or 0.45 μm nitrocellulose membranes (#1620115, Bio-Rad). Primary and secondary antibodies are listed in table S10. To induce proteostatic stress or block ISR activation, cells were treated with 1 μg/mL tunicamycin (T7765, Sigma) or 500 nM ISRIB (SML0843, Merck), respectively, for 3 hours.

### Proliferation and drug sensitivity assays

AML inducible knockdown cell lines were left untreated or treated with doxycycline (200 ng/mL) for 96 hours. Cells were then seeded in 96-well plates at 30,000 cells/mL and cultured for an additional 96 hours under the indicated treatment conditions, while maintaining knockdown induction. At least three technical replicates were included per condition. Cell proliferation was measured using an MTT assay (475989, Sigma). For drug sensitivity assays, cells were treated with a six-point, ten-fold dilution series of venetoclax, tunicamycin or ISRIB. For amino acid supplementation assays, cells were treated with a three-point, two-fold dilution series of two amino acid mixtures: RPQK, containing arginine (A5006, Sigma), proline (P5607, Sigma), glutamine (702596, Sigma) and lysine (L8662, Sigma); or AGMS, containing alanine (A7469, Sigma), glycine (G8790, Sigma), methionine (M5308, Sigma) and serine (S4311, Sigma). Cell growth was measured using the CellTiter-Blue Cell Viability Assay (G8081, Promega) according to the manufacturer’s instructions. Fluorescence was measured using a Varioskan LUX plate reader (Thermo Fisher Scientific).

### Immunofluorescence and confocal imaging

MOLM-13 cells were placed on poly-L-lysine-coated coverslips (P8920, Sigma-Aldrich) and incubated for 30 min at room temperature in PBS. Cells were pre-extracted and then fixed with 4% formaldehyde for 10 min at room temperature, followed by permeabilization with 0.2% Triton X-100 in PBS for 2 min at room temperature. Cells were blocked in 5% BSA in PBS for 30 min at room temperature. Primary antibodies were diluted in 1% BSA in PBS and incubated overnight at 4°C with agitation. After three washes with PBS containing 0.1% Tween-20, secondary antibodies diluted in 1% BSA in PBS were added for 30 min at room temperature protected from light. Cells were then washed three times with PBS containing 0.1% Tween-20 and incubated with DAPI for 1 min at room temperature. Coverslips were washed with PBS, dried and mounted onto microscope slides using VECTASHIELD mounting medium. Primary and secondary antibodies are listed in table S10. Images were acquired using a Leica SP5 confocal microscope equipped with an HCX PL APO lambda blue 63×/1.40 oil UV objective. The number of SRRM2-positive nuclear speckles per nucleus and the area of SC35-positive nuclear speckles were automatically quantified from four biological replicates using a custom ImageJ plug-in.

### Wright-Giemsa staining

MOLM-13 cells were placed on poly-L-lysine-coated 4-well plates and incubated for 30 min at room temperature in PBS. Cells were stained using Giemsa stain (WG16, Sigma) according to the manufacturer’s instructions. Images were acquired using an Olympus inverted microscope.

### RT-qPCR

Total RNA was purified using either the Direct-zol RNA MiniPrep Kit (R2050, Zymo Research) or the RNeasy Mini Kit (74104, Qiagen), according to the manufacturer’s instructions. For gene expression analysis, 500–1000 ng of total RNA were treated with RQ1 RNase-Free DNase (M6101, Promega) and reverse-transcribed using the Maxima H Minus First Strand cDNA Synthesis Kit (K1652, Thermo Fisher Scientific). cDNA samples were diluted and used as templates for RT-qPCR using iTaq Universal SYBR Green Supermix (1725124, Bio-Rad) on either a QuantStudio 5 Real-Time PCR System or a ViiA 7 Real-Time PCR System. Oligonucleotide sequences are listed in table S10.

### IP-MS assays

For IP-MS experiments, MOLM-13 cells were transduced with a lentiviral pHIV-ZsGreen construct expressing ALFA-tagged BCLAF1 or with the corresponding empty vector as an immunoprecipitation control. Transduced cells were isolated by flow cytometric sorting of ZsGreen-positive cells for both the empty vector and ALFA-BCLAF1 conditions. Protein extracts were prepared from 10 × 10^6^ cells. Cells were lysed in buffer containing 20 mM Tris-HCl pH 8.0 and 0.5% Triton X-100, supplemented with cOmplete protease inhibitor cocktail (11697498001, Sigma-Aldrich), PMSF (52332, Millipore), 2 mM MgCl_2_ and 2.5 kU benzonase (70746–4, Millipore). To promote nuclear lysis, 150 mM NaCl, 0.5 mM EDTA and 1 mM DTT were added. Protein concentration was determined using the Bradford assay, and 0.5 mg of total protein was used per immunoprecipitation after dilution in IP buffer consisting of 20 mM Tris-HCl pH 8.0, 0.5% Triton X-100, 150 mM NaCl, 0.5 mM EDTA and 1 mM DTT. For immunoprecipitation, ALFA Selector Resin (N1510, NanoTag Biotechnologies) was added to the protein extract and incubated for 2 hours at 4°C with rotation. Beads were washed four times with IP buffer and twice with 50 mM ammonium bicarbonate pH 8.0. Samples were then incubated overnight at 37°C with shaking at 1400 rpm in 50 mM ammonium bicarbonate pH 8.0 containing 250 ng sequencing-grade modified trypsin (Promega). Trypsin-digested peptides were separated from the beads by filtration through 0.45 μm Ultrafree-MC-HV spin columns (Merck-Millipore). Four biological replicates were performed. Digested peptides were acidified by adding trifluoroacetic acid (TFA) to a final concentration of 2%. Peptides were subsequently desalted and concentrated using triple-disc C18 StageTips assembled in-house from 200 μL micropipette tips and C18 matrix (Sigma-Aldrich). StageTips were activated with 100 μL methanol, followed by 100 μL Buffer B (80% acetonitrile, 0.1% formic acid) and 100 μL Buffer A (0.1% formic acid). Acidified peptide samples were loaded onto the StageTips, followed by two washes with 100 μL Buffer A. Peptides were eluted twice with 25 μL elution buffer containing 32.5% acetonitrile and 0.1% formic acid. Samples were vacuum-dried using a Universal Vacuum System UVS400S coupled to a SpeedVac SPD121P (Thermo Fisher Scientific) and stored at −20°*C. Prior* to mass spectrometry analysis, samples were reconstituted in 20 μL 0.1% formic acid and transferred to autosampler vials.

### LC-MS/MS data acquisition

Mass spectrometry data was acquired by nanoLC Easy 1000 (Proxeon, Odense, Denmark) coupled to a Q-Exactive Plus mass spectrometer (Thermo, Bremen, Germany). For the chromatography, peptides were separated in an HPLC EASY-Spray (Thermo Scientific) analytical column in a 60 min gradient from 2% to 33% acetonitrile gradient in 0.1% Formic Acid followed of 25 min of column re-equilibration. The mass spectrometer was operated in a Data-Dependent Acquisition mode with a top-15 method and a scan range of 400–1600 *m/z*. Full-scan MS spectra were acquired at a target value of 3 × 10^6^ and a resolution of 70,000, and the Higher-Collisional Dissociation (HCD) tandem mass spectra (MS/MS) were recorded at a target value of 5 × 10^5^ and with a resolution of 35,000, an isolation window of 2.2 m/z, and a normalized collision energy (NCE) of 25%. The minimum AGC target was 1 × 10^3^. The maximum MS1 and MS2 injection times were 20 and 120 ms, respectively. The precursor ion masses of scanned ions were dynamically excluded (DE) from MS/MS analysis for 60 s. Ions with charge 1, and >6, were excluded from triggering MS2 analysis.

### Mass spectrometry data analysis

All raw data was analyzed using MaxQuant (version 2.5.2.0) as previously described (*89*). Search was performed against an in-silico digested UniProt reference proteome for *Homo sapiens* including canonical and isoform sequences (29 August 2022). Database searches were performed according to standard settings with the following modifications: Digestion with Trypsin/P was used, allowing 2 missed cleavages. Oxidation (M), acetyl (protein N-term) allowed as variable modifications with a maximum number of 2. Carbamidomethyl (C) was disabled as fixed modification. Label-Free Quantification (LFQ) was enabled, not allowing Fast LFQ. Match between runs was enabled on default settings. Output from MaxQuant was exported and processed for statistical analysis in the Perseus computational platform version 1.6.14.0 (*90*). LFQ intensity values were log2 transformed and potential contaminants and proteins either identified by site or only reverse peptides were removed. Samples were grouped in experimental categories and proteins not identified in 4 out of 4 replicates in at least one group were removed. Missing values were imputed using normally distributed values with 0.3 width and 1.8 down shift for the total matrix. After imputation, statistical analysis was performed using two-sided Student’s *t* tests. Results were exported into in MS Excel 365 for a comprehensive browsing and visualization of the datasets. Volcano plots were constructed for data visualization using the VolcaNoseR web app (*91*).

### Metabolomics

MOLM-13 inducible knockdown cell lines (shCONTROL, shBCLAF1 and shATF4 #1) were treated with doxycycline for 96 h in standard RPMI culture medium. Cells were then pelleted, washed, and seeded in glucose-free RPMI medium (11879020) supplemented with 11 mM U-^13^C_6_-glucose (CLM-1396-2, Cambridge Isotope Laboratories) for 24 h. Cells were subsequently quenched and metabolites were extracted as previously described (*92*). Briefly, cells were rapidly pelleted by centrifugation for 1 min at 1500g, followed by two quick washes with −40°C quenching buffer consisting of 10 mM ammonium acetate in 60:40 methanol. Quenched cell pellets were frozen on dry ice and stored at −80°C until extraction. Metabolite extraction was performed using cold two-phase methanol–water–chloroform extraction containing 0.75 mg/mL glutaric acid (Sigma-Aldrich) as an internal standard. After vortexing for 10 min, phase separation was achieved by centrifugation at 4°C for 10 min. The methanol phase, containing polar metabolites, was collected and dried using a nitrogen evaporator. For metabolite detection and quantification, gas chromatography–mass spectrometry (GC-MS) analysis was performed as previously described (*93*). Briefly, the polar metabolite fraction was derivatized with 20 μL of 20 mg/mL methoxyamine in pyridine for 90 min at 37°C. Subsequently, 15 μL of N-(tert-butyldimethylsilyl)-N-methyl-trifluoroacetamide containing 1% tert-butyldimethylchlorosilane were added to 7.5 μL of each derivative and incubated for 60 min at 60°C. Metabolites were separated by gas chromatography using an Agilent 8860 GC system, with the inlet temperature set at 270°C and 1 μL splitless sample injection on a DB35MS column (30 m, 0.25 mm, 0.25 μm). Elution was performed using helium as the carrier gas at a constant flow rate of 1 mL/min and a temperature gradient. The column temperature was initially held at 100°C for 1 min, increased at 2.5°C/min to 105°C and held for 2 min, then increased at 3.5°C/min to 240°C, and finally increased at 3.5°C/min to 320°C and held for 2 min. After elution, polar metabolites were detected using an Agilent 5977C MS system with electron impact ionization. Source and quadrupole temperatures were set at 230°C and 150°C, respectively. Peak areas were normalized to protein content or total cell number and to the internal standard glutaric acid. MATLAB was used to extract mass distribution vectors and integrated raw ion chromatograms. Natural isotope distributions were corrected using theoretical mass distribution vectors (MDVs) for all potentially labeled isotopologues of each metabolite (*94*).

### RNA-seq

MOLM-13 inducible knockdown cell lines (shCONTROL and shBCLAF1) were treated with doxycycline for 96 hours. RNA was extracted using the Direct-zol RNA MiniPrep Kit (R2050, Zymo Research), according to the manufacturer’s instructions. Three independent biological replicates were collected for each condition. RNA libraries were prepared and sequenced at the CABIMER Genomics Facility (CABIMER, Sevilla). Libraries were prepared using the Stranded Total RNA Prep with Ribo-Zero kit (Illumina), and sequencing was performed using the NovaSeq 6000 SP Reagent Kit (Illumina) with 2 × 50 bp paired-end reads on a NovaSeq 6000 system (Illumina).

### RIPseq and RIP-qPCR

MOLM-13 inducible knockdown cell lines were collected after 96 hours of doxycycline treatment for BCLAF1 RIP-seq using an anti-BCLAF1 antibody. Cell pellets were lysed in PEB buffer consisting of 20 mM Tris-HCl pH 7.5, 100 mM KCl, 5 mM MgCl_2_, 10% NP-40, cOmplete protease inhibitor cocktail (11697498001, Sigma-Aldrich), PMSF (52332, Millipore), and 5 μL/mL RNasin Ribonuclease Inhibitor (N2111, Promega). Immunoprecipitation was performed in NT2 buffer consisting of 50 mM Tris-HCl pH 7.5, 150 mM NaCl, 1 mM MgCl_2_ and 0.05% NP-40, supplemented with 1 mM DTT, RNasin, protease inhibitors and 1.5 mM EDTA, using protein extract adjusted to a final volume of 1 mL. For each immunoprecipitation, 2 μg of anti-BCLAF1 antibody were used. Antibodies were first coupled to protein A/G Dynabeads for 1 h at room temperature with rotation. Beads were then washed with NT2 buffer, added to the protein lysate, and incubated overnight at 4°C with rotation. DNase treatment (AM1907, Invitrogen) was performed on beads for 10 min at 37°C. RNA was eluted from beads and proteins were digested by treatment with Proteinase K (25530049, Thermo Fisher Scientific) for 20 min at 55°C with agitation. RNA was purified from the eluate using TRIzol according to the manufacturer’s instructions. RNA was then either used for library preparation for RIP-seq, as described for RNA-seq, or reverse-transcribed using the Maxima H Minus First Strand cDNA Synthesis Kit (K1652, Thermo Fisher Scientific) for RIP-qPCR analysis. Ten percent of the protein extract used for immunoprecipitation was processed in parallel as input using TRIzol extraction.

### RNA-seq data analysis

Reads were aligned using STAR v2.7.11b (*95*) and mapped to hg38 reference human genome. Quantification of the reads was performed using StringTie v2.2.3 (*96*). Downstream analysis of the data was conducted with custom Rscripts (https://github.com/gmzlab/BCLAF1_MOLM13), under R v4.3.2. Differential gene expression analysis was performed using DESeq2 v1.42.1 from Bioconductor (*97*). Differentially expressed genes were defined using an absolute FC ≥ 2 and FDR < 0.05. For RIP-seq data, BCLAF1-bound RNAs were identified using DESeq2 with FC ≥ 1.5 and FDR < 0.05. Gene ontology (GO) enrichment analysis and gene set enrichment analysis (GSEA) were performed using clusterProfiler v4.10.1 (*98*). Significant GO terms were defined using FDR < 0.05. Venn diagrams were generated using the R package VennDiagram v1.7.3.

### Publicly available data analysis

RNA-seq data from TCGA cohorts was downloaded using TCGAbiolinks v2.30.4 (*99*). Downstream analyses were performed using a custom Rscript which uses the same packages as the one used for RNA-seq (https://github.com/gmzlab/BCLAF1_MOLM13). Differentially expressed genes and gene ontology terms were determined by the same thresholds fixed in our RNA-seq data. The cohorts used were: BEAT-AML (*30*) and TARGET-AML (*100*). ENCODE ChIP-seq datasets for ATF4 in K562 cells were downloaded from the GEO (GSE127432). Reads were mapped using BWA v0.7.17 (*101*), downstream analysis samtools v1.13 and peak calling using MACS3 v3.0.2 (*102*).

### Statistical analysis

All data in bar and line plots are presented as mean ± SEM from at least three independent replicates, unless stated otherwise. Statistical significance was assessed using two-way ANOVA, unpaired Student’s *t* tests or paired Student’s *t* tests, as indicated. All statistical analyses were performed using GraphPad Prism. Only statistically significant differences are shown.
