# Supplementary material for: BCLAF1 links RNA splicing to ATF4-dependent metabolic adaptation in acute myeloid leukemia
Source: Sci Adv. 2026 Nov 4;12(45):eaeg0571. doi: 10.1126/sciadv.aeg0571 (PMC13743010; doi:10.1126/sciadv.aeg0571)
Supplement: Supplementary file 1 [file sciadv.aeg0571_sm.pdf]

Science Advances
AAAS

Supplementary Materials for

BCLAF1 links RNA splicing to ATF4-dependent metabolic adaptation in acute myeloid leukemia

Laura López-Hernández *et al.*

Corresponding author: Jeffrey J Bednarski, jeb683@pitt.edu;
Gonzalo Millán-Zambrano, gonzalo.millan@cabimer.es

Sci. Adv. **12**, eaeg0571 (2026)
DOI: 10.1126/sciadv.aeg0571

The PDF file includes:

Figs. S1 to S7
Legends for tables S1 to S10

Other Supplementary Material for this manuscript includes the following:

Tables S1 to S10

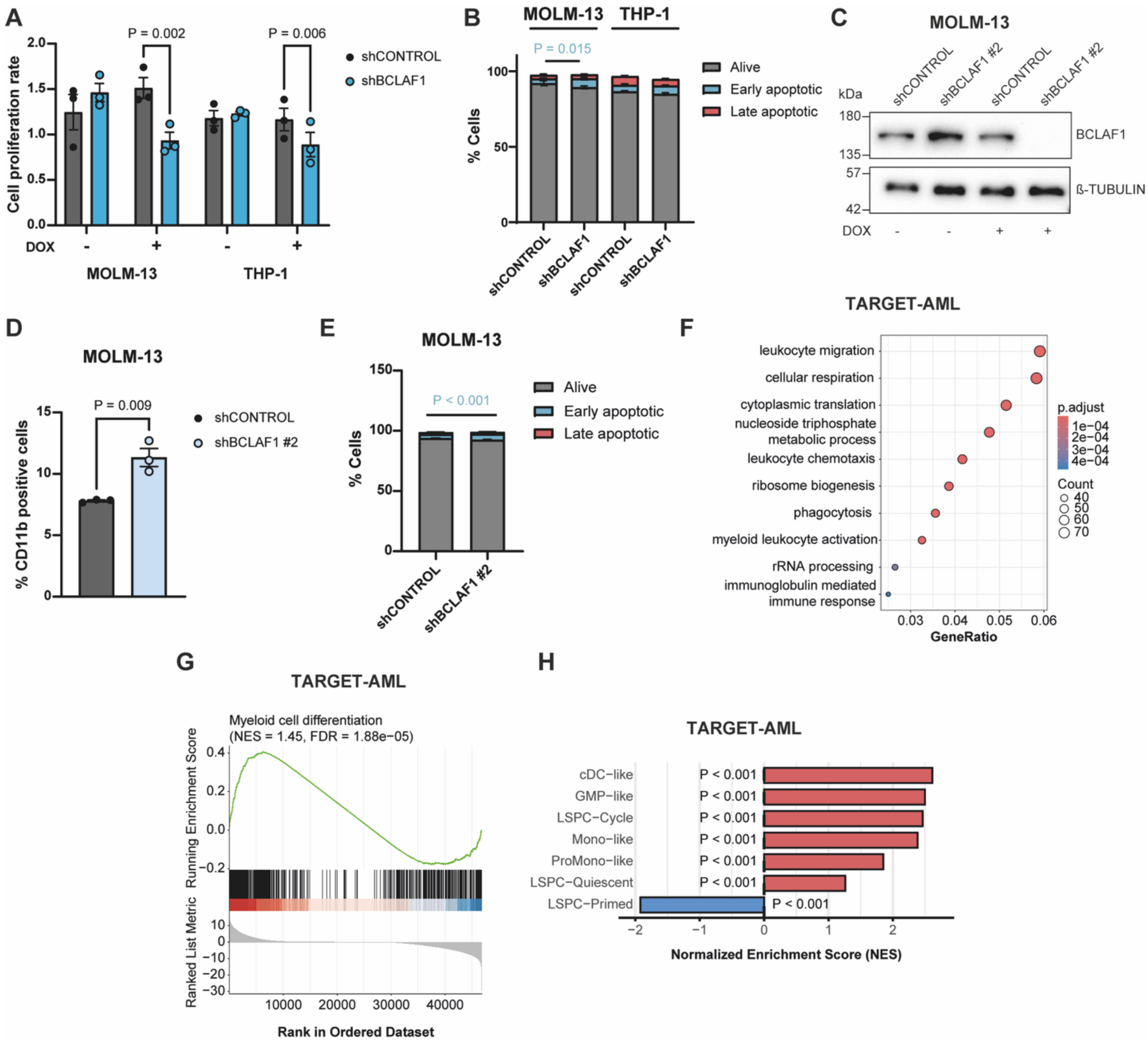

fig. S1. Supplementary to BCLAF1 supports AML pathogenesis. (**A**) Cell proliferation assay (MTT-based) in shCONTROL and shBCLAF1 cells treated or not with DOX for 96 hours, followed by 96 additional hours in culture. Data represent mean ± SEM of three biological replicates. Statistics by paired t-test. (**B**) Annexin V/PI flow cytometry analysis showing the distribution of alive, early apoptotic and late apoptotic cells following 96h of DOX treatment. Data represent mean ± SEM of three biological replicates. Statistics by paired t-test. (**C**) Immunoblot analysis of BCLAF1 protein levels in shCONTROL and shBCLAF1 #2 cells treated or not with DOX for 96 hours. β-tubulin was used as a loading control. (**D**) Flow cytometry analysis showing the percentage of CD11b-positive cells in shCONTROL and shBCLAF1 #2 cells treated with DOX for 96h. Data represent mean ± SEM of three biological replicates. Statistics by unpaired t-test. (**E**) Annexin V/PI flow cytometry analysis showing the distribution of alive, early apoptotic and late apoptotic cells following 96h of DOX treatment. Data represent mean ± SEM of three biological replicates. Statistics by unpaired t-test. (**F**) Dot plot of GO terms enriched among genes up-regulated in BCLAF1-low versus BCLAF1-high patient samples from the TARGET-AML cohort. GeneRatio indicates the proportion of significantly upregulated genes associated with each

GO term. Dot size reflects the number of genes per term; color denotes adjusted p-value. (**G**) Gene set enrichment analysis (GSEA) of myeloid cell differentiation–related gene sets in BCLAF1-low versus BCLAF1-high AML patient samples from the TARGET-AML cohort. (**H**) Gene set enrichment analysis (GSEA) of the indicated gene sets in BCLAF1-low versus BCLAF1-high AML patient samples from the TARGET-AML cohort.

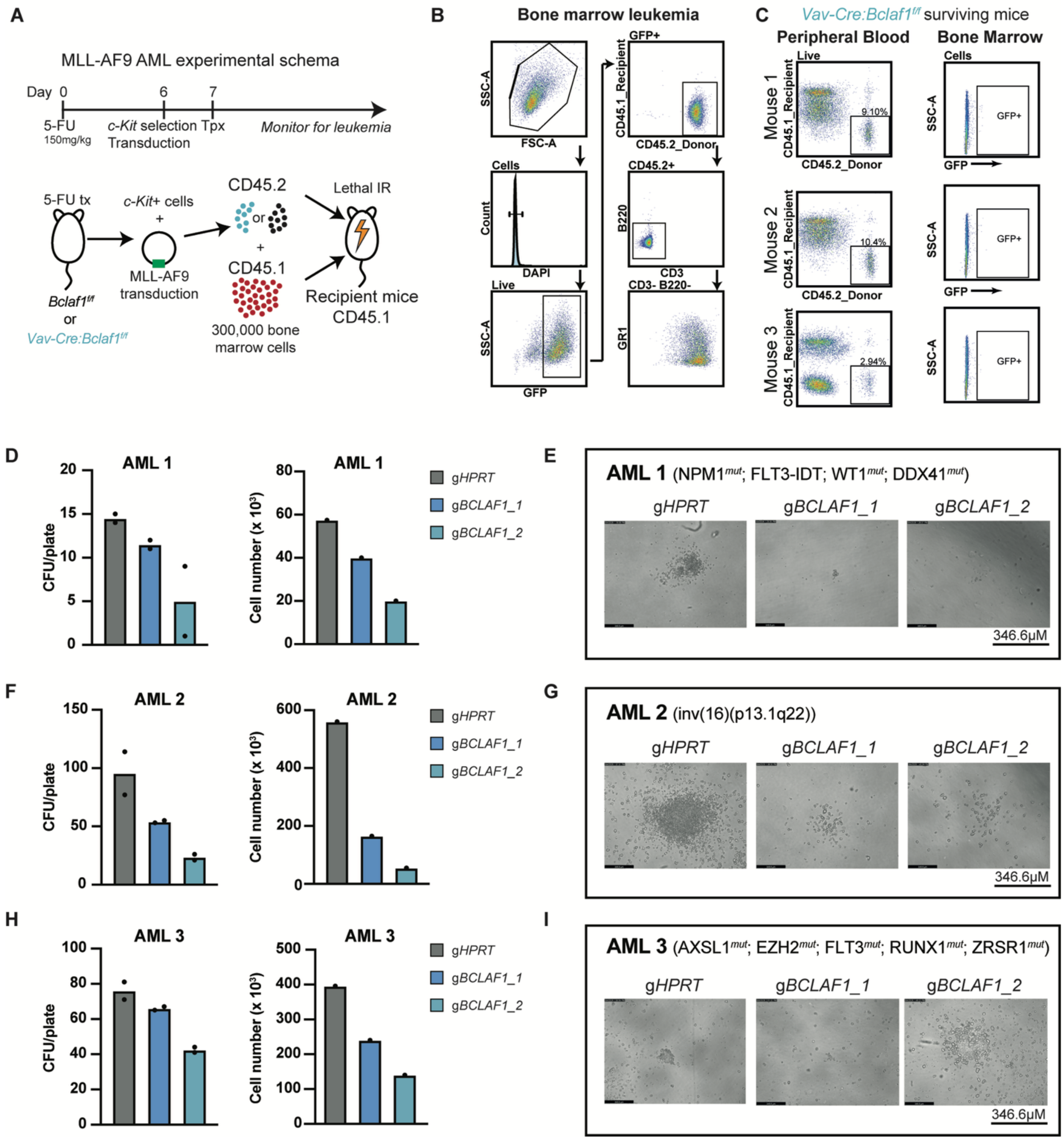

fig. S2. Supplementary to BCLAF1 supports AML pathogenesis. (**A**) Schematic of *in vivo* leukemia experiments in Figure 1J using mice with hematopoietic-specific loss of *Bclaf1*. HSPCs from CD45.2+ *Bclaf1*$^{f/f}$ or *Vav-Cre:Bclaf1*$^{f/f}$ mice were transduced with retrovirus co-expressing MLL-AF9 and GFP then transplanted with 300,000 CD45.1+ recipient bone marrow cells into lethally-irradiated CD45.1+ recipient mice. Leukemic burden was assessed by percentage of GFP+ cells in peripheral blood. Mice were euthanized once peripheral blood showed ≥ 80% leukemia. (**B**) Representative flow cytometry gating for bone marrow of mice with leukemia from Figure 1J. Leukemia cells were defined as GFP+. (**C**) Flow cytometry of peripheral blood and bone marrow from representative surviving mice that received CD45.2+ *Vav-Cre:Bclaf1*$^{f/f}$ HSPCs transduced

with MLL-AFP- and GFP-expressing retrovirus. Peripheral blood shows percentage of CD45.2+ donor cells in all live cells. Bone marrow shows percentage of GFP+ leukemia cells among all leukocytes. Data are from 3 independent mice that were alive at 154 days post-transplant in Figure 1J. (**D**), (**E**), (**F**), (**G**), (**H**) and (**I**) Primary AML cells were collected directly from the bone marrow or peripheral blood of three independent patients. AML1 harbored mutations in NPM1, FLT3-ITD, WT1 and DDX41. AML2 carried inv(16). AML3 was a secondary AML arising after progression from myelodysplastic syndrome and harbored mutations in ASXL1, EZH2, FLT3, RUNX1 and ZRSR2. Freshly collected AML cells were transfected with Cas9/gRNA ribonucleoprotein (RNP) complexes targeting either *HPRT* as a control (*sgHPRT*) or *BCLAF1* using two independent sgRNAs (*sgBCLAF1_1* and *sgBCLAF1_2*). Cells were subsequently plated in MethoCult. (**D**), (**F**) and (**H**) Colony-forming units (CFUs) and total cell numbers were quantified 10 days after plating. CFU data represent mean from two technical replicates per sgRNA. Cell numbers correspond to the total number of cells recovered from all CFUs across both technical replicates per gRNA. (**E**), (**G**) and (**I**) Representative images of CFUs are shown.

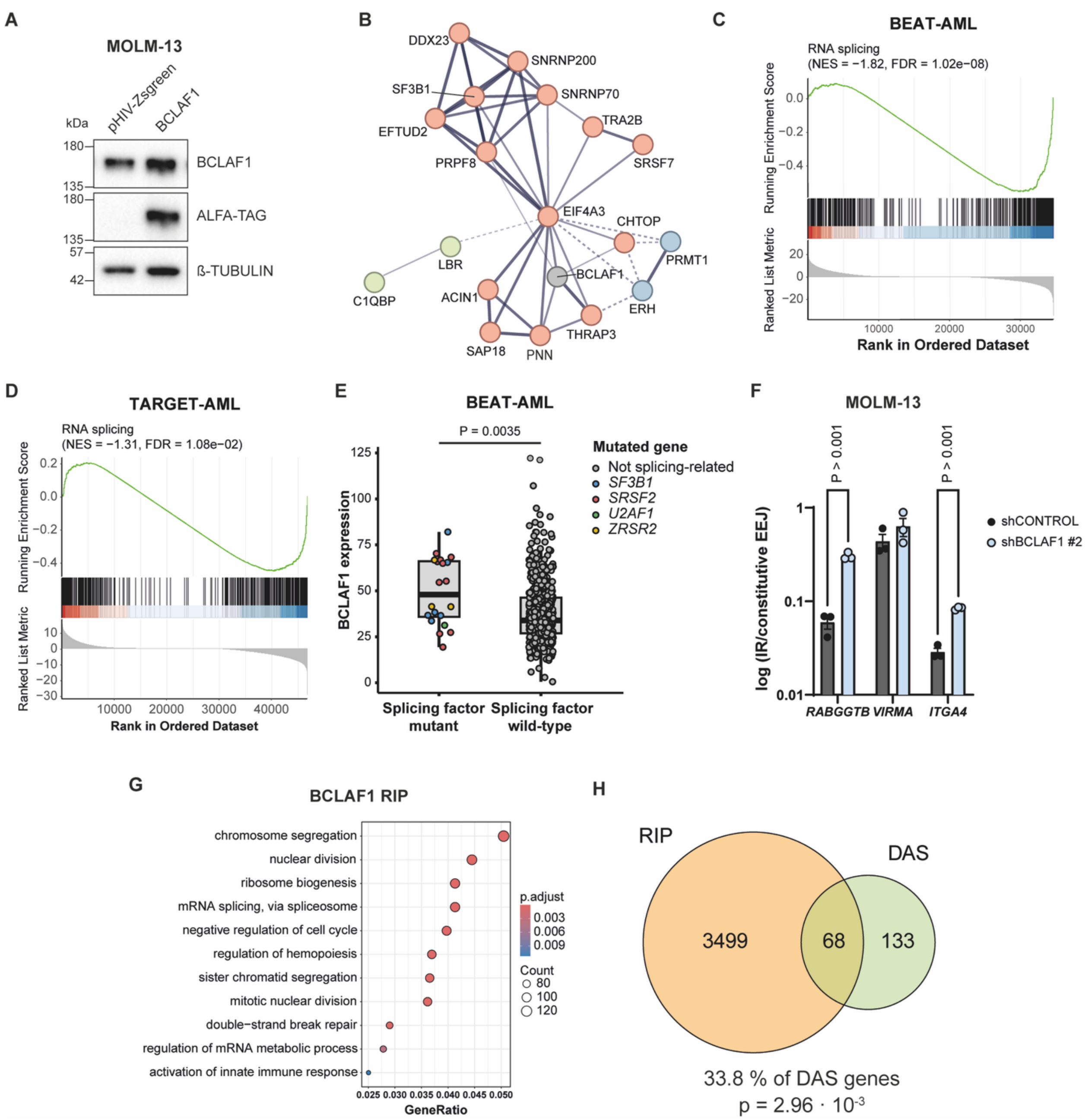

fig. S3. Supplementary to BCLAF1 regulates mRNA splicing in AML cells. (**A**) Immunoblot analysis of MOLM-13 cells infected with a lentiviral vector expressing ALFA-tagged BCLAF1 or empty vector control (pHIV-Zsgreen). Blots were probed with anti-BCLAF1 and anti-ALFA antibodies. β-tubulin was used as a loading control. (**B**) STRING network analysis of BCLAF1 interactors (n = 19) identified in panel A. Grey circle indicates BCLAF1; red circles represent spliceosome components. (**C**) and (**D**) Gene set enrichment analysis (GSEA) of RNA splicing–related gene sets in BCLAF1-low versus BCLAF1-high AML patient samples from the BEAT-AML and TARGET-AML cohorts, respectively. (**E**) Box plot showing BCLAF1 mRNA levels in BEAT-AML patient samples harboring at least one mutation in a splicing factor-related gene (SF3B1, SRSF2, U2AF1 or ZRSR2) compared with samples without mutations in any of these

genes. Statistical significance was assessed using a one-sided Wilcoxon rank-sum test. (**F**) RT-qPCR experiments showing relative intron retention (IR) levels of the indicated transcripts in shCONTROL and shBCLAF1 #2 MOLM-13 cells treated with DOX for 96 hours. Data represent mean ± SEM of three biological replicates. Statistics by unpaired t-test. (**G**) Dot plot of GO terms enriched among BCLAF1 interactors. Dot size reflects the number of genes per term; color denotes adjusted p-value. (**H**) Venn diagram showing the overlap between BCLAF1-bound transcripts (RIP) and transcripts with differential alternative splicing (DAS) upon BCLAF1 knockdown.

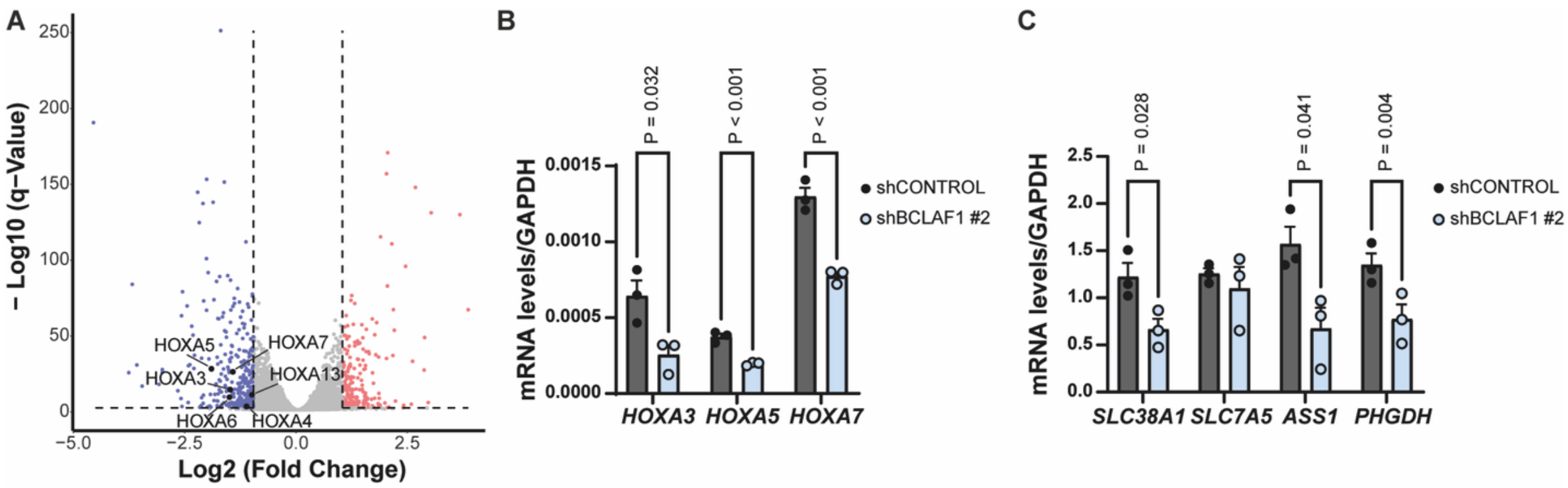

fig. S4. Supplementary to BCLAF1 promotes amino acid metabolic gene expression. (**A**) Volcano plot showing differentially expressed genes in shBCLAF1 versus shCONTROL MOLM-13 cells treated with DOX for 96 hours. The x-axis indicates log2 fold change and the y-axis represents –log10 adjusted p-value. Significantly up-regulated (red) and down-regulated (blue) genes are shown (fold change ≥ 2, adjusted p ≤ 0.05). (**B**) and (**C**) RT-qPCR experiments showing relative mRNA levels of the indicated transcripts in shCONTROL and shBCLAF1 #2 MOLM-13 cells treated with DOX for 96 hours. Data were normalized to GAPDH and represent mean ± SEM of three biological replicates. Statistics by unpaired t-test.

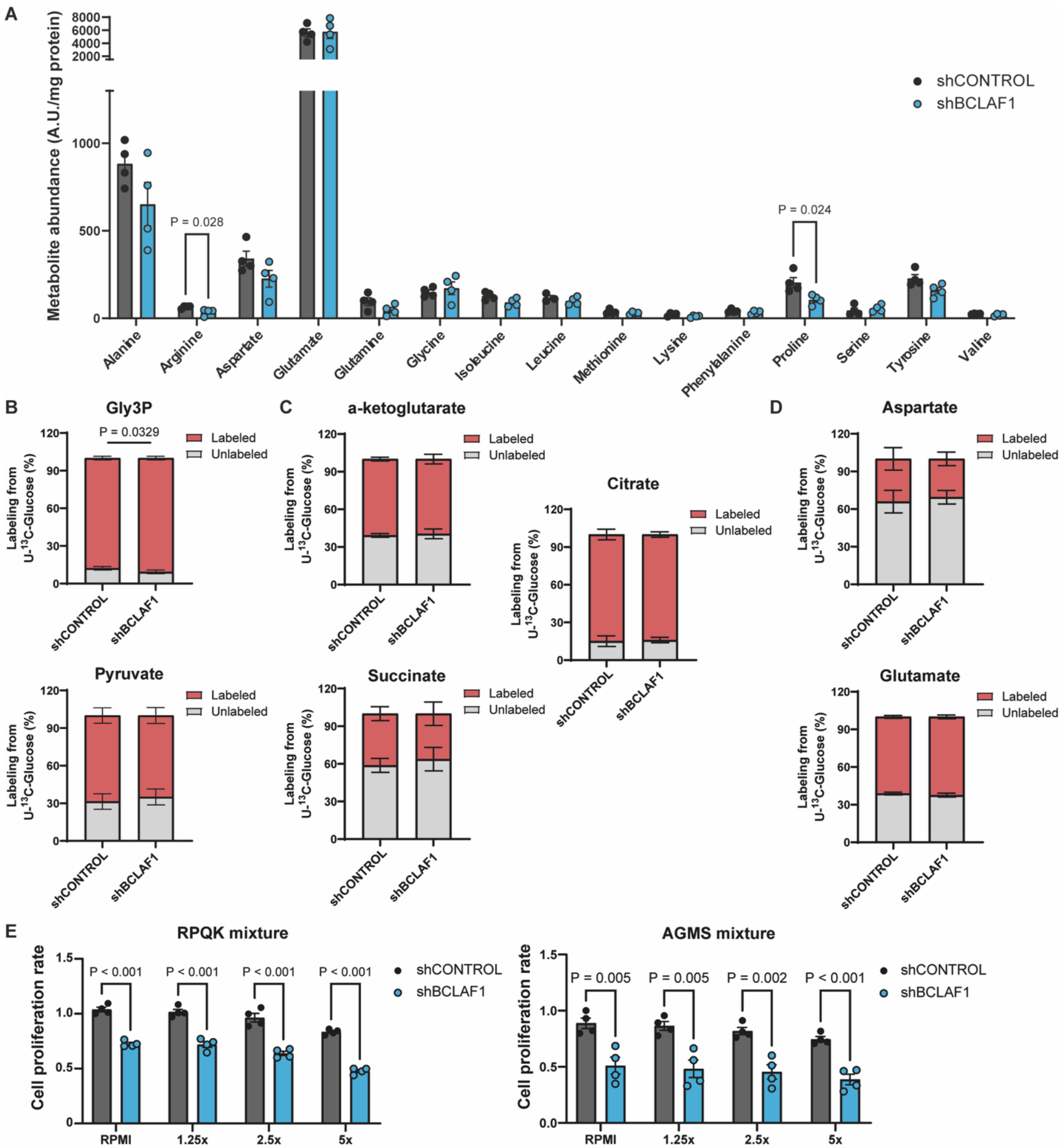

fig. S5. Supplementary to loss of BCLAF1 disrupts *de novo* amino acid synthesis. (A) Bar plot showing total amino acid levels in shCONTROL and shBCLAF1 MOLM-13 cells treated with DOX for 96 hours. Data are shown as mean ± SEM of four technical replicates from one representative experiment. Statistics by unpaired t-test. The experiment was independently repeated with similar results. The experiment was independently repeated with similar results. (**B**), (**C**) and (**D**) Percentage of unlabeled (M+0; gray) or labeled (the sum of M+1, M+2, and M+3; red) metabolite levels. Data are shown as mean ± SEM of four technical replicates from one representative experiment Statistics by two-way ANOVA. The experiment was independently repeated with similar results. (**E**) Cell proliferation assay (CellTiter-Blue-based) in shCONTROL

and shBCLAF1 MOLM-13 cells treated with DOX for 96 hours, followed by 96 additional hours in culture supplemented with increasing levels of two amino acid mixtures. Amino acids were added at 1×, 1.25×, 2.5× and 5× their standard RPMI concentrations. Data represent mean ± SEM of three biological replicates. Statistics by paired t-test.

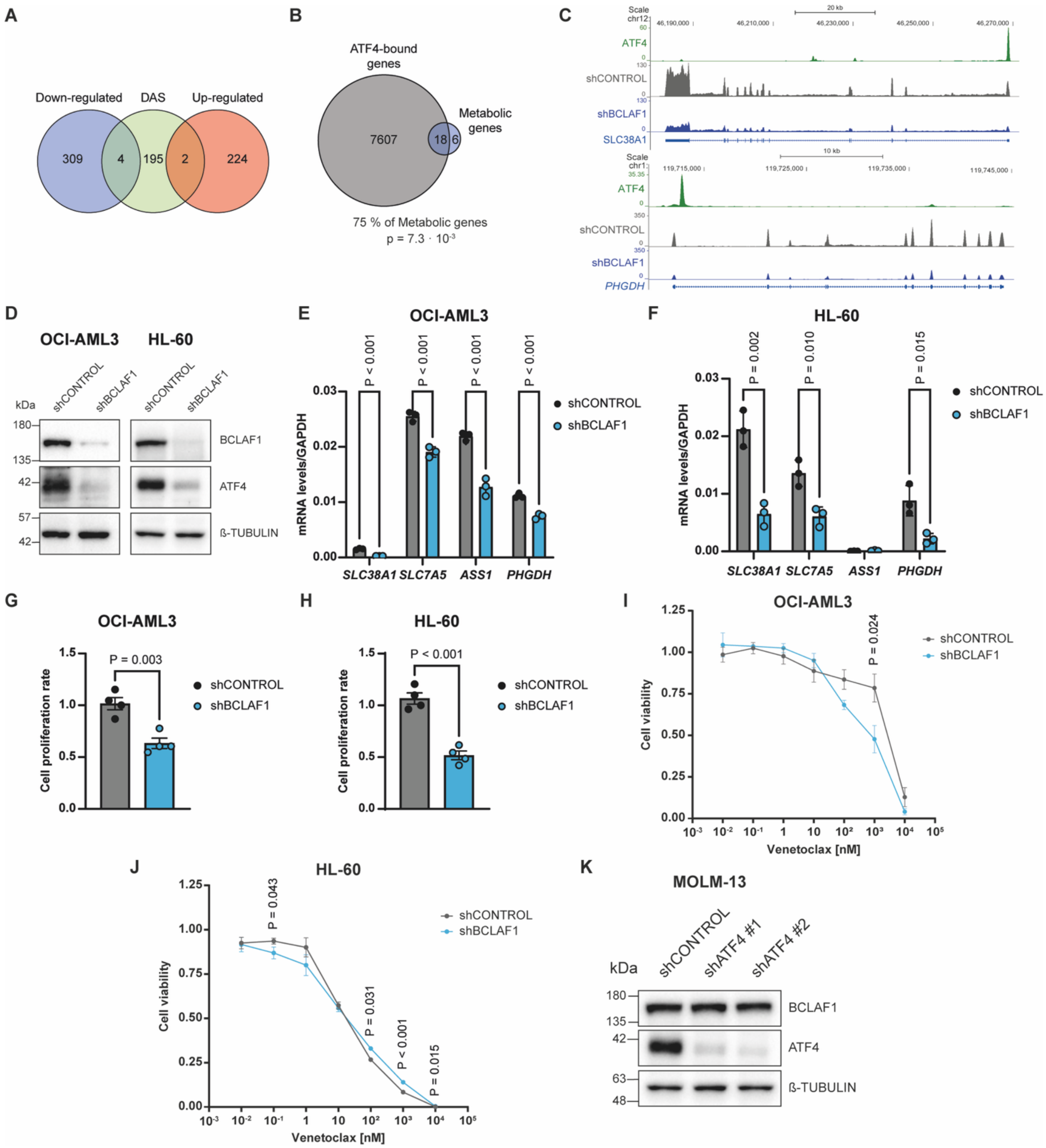

fig. S6 Supplementary to BCLAF1 supports amino acid metabolism through ATF4. (**A**) Venn diagram showing the overlap between up-regulated, down-regulated and differentially spliced genes (DAS) upon BCLAF1 depletion. (**B**) Venn diagram showing the overlap between genes downregulated upon BCLAF1 depletion (MOLM-13 RNA-seq) and genes with significant ATF4 ChIP-seq peaks (ENCODE, K562 cells). Only genes annotated in amino acid metabolism-related GO categories were considered among the downregulated set. Overlap significance was assessed by Fisher's exact test. (**C**) Genome browser snapshots showing ATF4 ChIP-seq signal from ENCODE data in K562 cells together with RNA-seq data from shCONTROL and shBCLAF1 MOLM-13 cells treated with DOX for 96 hours. (**D**) Immunoblot analysis of BCLAF1 and ATF4

protein levels in shCONTROL and shBCLAF1 cells treated with DOX for 96 hours. β-tubulin was used as a loading control. (**E**) and (**F**) RT-qPCR experiments showing relative mRNA levels of the indicated transcripts in shCONTROL and shBCLAF1 cells treated with DOX for 96 hours. Data were normalized to *GAPDH* and represent mean ± SEM of three biological replicates Statistics by unpaired t-test. (**G**) and (**H**) Cell proliferation assay (CellTiter-Blue-based) in shCONTROL and shBCLAF1 cells treated with DOX for 96 hours, followed by 96 additional hours in culture. Data represent mean ± SEM of four biological replicates. Statistics by unpaired t-test. (**I**) and (**J**) Cell viability assay (CellTiter-Blue-based) in shCONTROL and shBCLAF1 cells treated with DOX for 96 hours, followed by 96 additional hours of Venetoclax treatment. Data is normalized to DMSO control and represent mean ± SEM of four biological replicates. Statistics by unpaired t-test. (**K**) Immunoblot analysis of BCLAF1 and ATF4 protein levels in shCONTROL and shATF4 #1 and #2 cells treated with DOX for 96 hours. β-tubulin was used as a loading control.

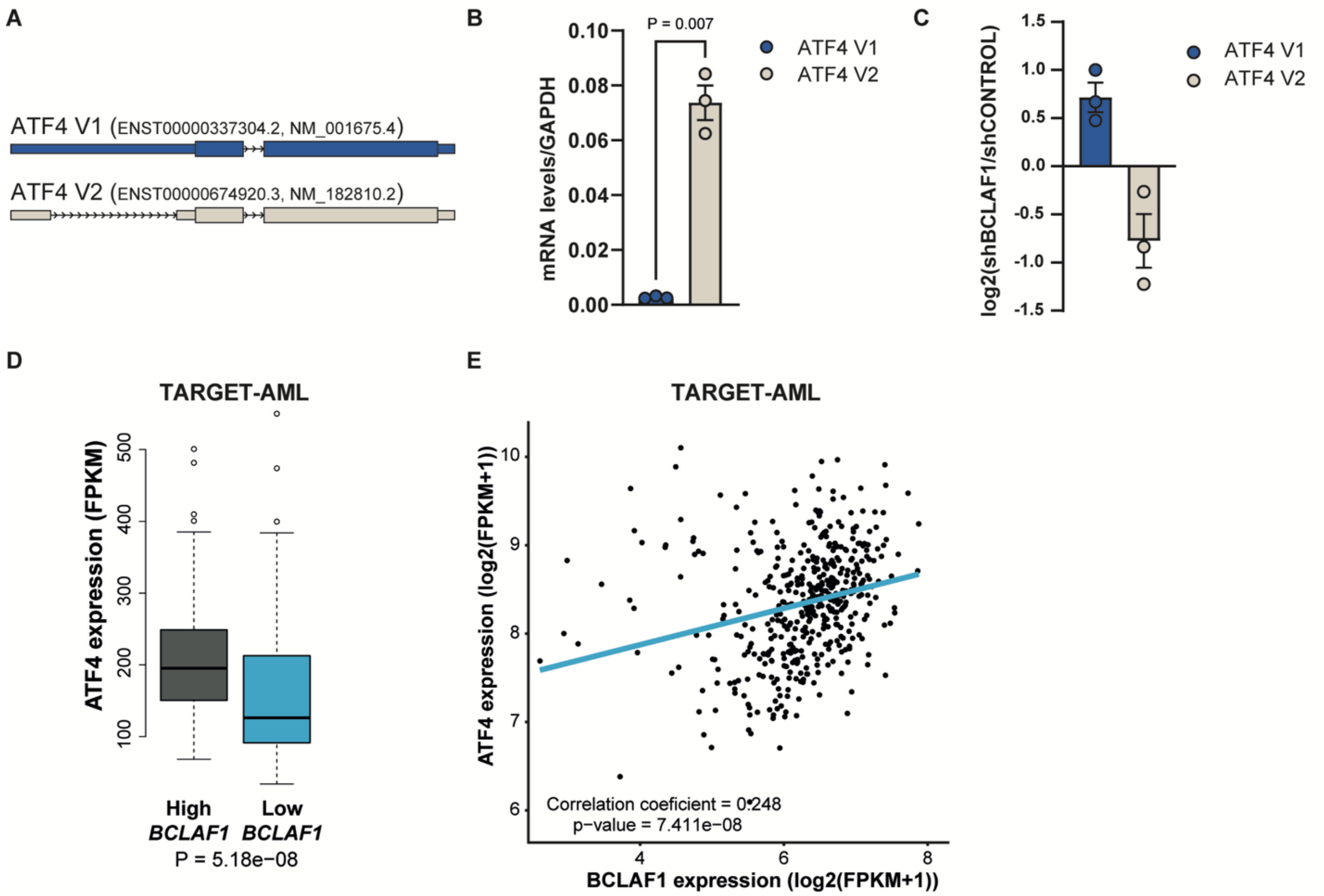

fig. S7 Supplementary to BCLAF1 controls *ATF4* mRNA alternative splicing. (**A**) Schematic representation of both ATF4 isoforms being V1 the one that has an intron retention and V2 the functional one. (**B**) RT-qPCR experiments showing relative mRNA levels of the indicated transcripts in shCONTROL MOLM-13 cells treated with DOX for 96 hours. Data were normalized to *GAPDH* and represent mean ± SEM of three biological replicates. Statistics by unpaired t-test. (**C**) RT-qPCR experiments showing the ratio of relative mRNA levels of the indicated transcripts between shBCLAF1 and shCONTROL MOLM-13 cells treated with DOX for 96 hours. Data were normalized to *GAPDH* and represent+ log2 of the ratio from both samples and ± SEM. (**D**) Box plot showing ATF4 mRNA levels in BCLAF1-high (top 25%) and BCLAF1-low (bottom 25%) patient samples from the TARGET-AML cohort. Statistical significance was assessed using Wilcoxon Rank Sum and Signed Rank Tests (one-sided). (**E**) Scatter plot showing the correlation between BCLAF1 and ATF4 mRNA levels across TARGET-AML patient samples. Pearson correlation coefficient (r) and p-value are indicated.

Table S1. Differential gene expression results from BEAT-AML cohort data.
See Excel spreadsheet.

Table S2. Differential gene expression results from TARGET-AML cohort data.
See Excel spreadsheet.

Table S3. Mass spectrometry results from BCLAF1 immunoprecipitation (IP-MS).
See Excel spreadsheet.

Table S4. Splicing analysis results upon BCLAF1 depletion using MAJIQ.
See Excel spreadsheet.

Table S5. Transcriptome-wide RNA binding analysis by BCLAF1 RIP-seq.
See Excel spreadsheet.

Table S6. Differential gene expression results from BCLAF1 MOLM-13 deficient cells.
See Excel spreadsheet.

Table S7. Metabolomic results upon BCLAF1 depletion for total metabolites and isotope tracing experiments.
See Excel spreadsheet.

Table S8. Transcriptional regulatory networks analysis results using downregulated genes upon BCLAF1 depletion using TTRUST.
See Excel spreadsheet.

Table S9. Metabolomic results upon ATF4 depletion for isotope tracing experiments.
See Excel spreadsheet.

Table S10. Full list of used oligos and antibodies.
See Excel spreadsheet.
